# Assessing Temporal and Spatial Patterns of Observed and Predicted Ozone in Multiple Urban Areas

**DOI:** 10.1289/EHP190

**Published:** 2016-05-06

**Authors:** Heather Simon, Benjamin Wells, Kirk R. Baker, Bryan Hubbell

**Affiliations:** Office of Air Quality Planning and Standards, U.S. Environmental Protection Agency, Research Triangle Park, North Carolina, USA

## Abstract

**Background:**

Ambient monitoring data show spatial gradients in ozone (O3) across urban areas. Nitrogen oxide (NOx) emissions reductions will likely alter these gradients. Epidemiological studies often use exposure surrogates that may not fully account for the impacts of spatially and temporally changing concentrations on population exposure.

**Objectives:**

We examined the impact of large NOx decreases on spatial and temporal O3 patterns and the implications on exposure.

**Methods:**

We used a photochemical model to estimate O3 response to large NOx reductions. We derived time series of 2006–2008 O3 concentrations consistent with 50% and 75% NOx emissions reduction scenarios in three urban areas (Atlanta, Philadelphia, and Chicago) at each monitor location and spatially interpolated O3 to census-tract centroids.

**Results:**

We predicted that low O3 concentrations would increase and high O3 concentrations would decrease in response to NOx reductions within an urban area. O3 increases occurred across larger areas for the seasonal mean metric than for the regulatory metric (annual 4th highest daily 8-hr maximum) and were located only in urban core areas. O3 always decreased outside the urban core (e.g., at locations of maximum local ozone concentration) for both metrics and decreased within the urban core in some instances. NOx reductions led to more uniform spatial gradients and diurnal and seasonal patterns and caused seasonal peaks in midrange O3 concentrations to shift from midsummer to earlier in the year.

**Conclusions:**

These changes have implications for how O3 exposure may change in response to NOx reductions and are informative for the design of future epidemiology studies and risk assessments.

**Citation:**

Simon H, Wells B, Baker KR, Hubbell B. 2016. Assessing temporal and spatial patterns of observed and predicted ozone in multiple urban areas. Environ Health Perspect 124:1443–1452; http://dx.doi.org/10.1289/EHP190

## Introduction

Exposure to ozone (O_3_) is known to cause negative health effects in humans [U.S. Environmental Protection Agency (EPA) 2013]. Many areas in the United States currently experience O_3_ concentrations that exceed the National Ambient Air Quality Standards (NAAQS) (https://ozoneairqualitystandards.epa.gov/OAR_OAQPS/OzoneSliderApp/index.html#). Although there have been substantial emission reductions of O_3_ precursors, and peak O_3_ concentrations have decreased as a result of these reductions, some areas are expected to continue to exceed the 8-hr O_3_ NAAQS in the future (U.S. EPA 2014b).

Nitrogen oxide (NO_x_) and volatile organic compound (VOC) emissions react in the atmosphere through complex nonlinear chemical reactions to form ground-level O_3_ when meteorological conditions are favorable (Seinfeld and Pandis 2012). Specifically, NO_x_ participates in chemical pathways for both O_3_ formation and destruction; therefore, the net impact of NO_x_ emissions on O_3_ concentrations depends on the relative abundances of NO_x_, VOCs, and sunlight as well as on the temporal and spatial scales being examined. Emissions sources of NO_x_ and VOC and the resulting O_3_ vary seasonally, diurnally, and spatially. Spatial differences exist from rural to urban, city to city, and even within a given urban area (Marshall et al. 2008). In locations with high concentrations of nitric oxide (NO), one component of NO_x_, O_3_ can become artificially suppressed owing to direct reactions of NO with O_3_. These reactions also result in the production of other oxidized nitrogen species, which form O_3_ away from the emission source location while the wind transports the air mass, thus contributing to elevated O_3_ downwind (Cleveland and Graedel 1979). As a result of this chemistry, decreasing NO_x_ and VOC emissions generally decrease O_3_ at times and locations in which O_3_ concentrations are high (Simon et al. 2013). In limited circumstances, reductions of NO_x_ emissions can lead to O_3_ increases in the immediate vicinity of highly concentrated NO_x_ sources, whereas these same emissions changes generally lead to reductions of O_3_ downwind over longer timescales (Cleveland and Graedel 1979; Kelly et al. 2015; Murphy et al. 2007; Sillman 1999; Xiao et al. 2010). Because emissions of both NO_x_ and VOC decrease from sources that have unique temporal and spatial attributes (e.g., power plant NO_x_ and mobile source NO_x_ and VOCs), it will be important to characterize how O_3_ formation regimes vary over different temporal and spatial scales in order to understand how O_3_ concentrations may change as a result of these emissions reductions.

Spatial and temporal variability in pollutant concentrations are key inputs to studies evaluating associations between air quality and human health. In the past, epidemiological studies linking O_3_ with human health impacts have used ambient measurements in various ways to match against health outcomes. Some studies have used spatially averaged O_3_ concentrations for an entire urban area to link with short-term (Smith et al. 2009; Zanobetti and Schwartz 2008) and long-term (Jerrett et al. 2009) mortality. Other studies have used the O_3_ monitor closest to the residence of the human subjects (Bell 2006). The use of an average “composite” monitor masks both spatial and temporal heterogeneity that exist in an urban area. Using the nearest monitor may provide a better spatial match but does not fully consider daily activity patterns. Given the spatial and temporal heterogeneity in O_3_ and human activity patterns that place a subject in different places at different times of the day, it is potentially important to match activity with a realistic heterogeneous representation of O_3_ concentrations. This is true for conducting epidemiology studies of O_3_ health outcomes, as well as in the application of the results of those studies in risk and health impact assessments. To the extent that reductions in NO_x_ and VOC emissions change the temporal and spatial patterns of O_3_, the use of a spatially averaged O_3_ concentration may mask how population-weighted O_3_ exposures change.

Recent risk assessments conducted to inform the U.S. EPA review of the O_3_ NAAQS included application of results from both controlled human exposure studies and observational epidemiology studies (U.S. EPA 2014a). Estimates of risk based on controlled human exposure studies evaluated changes in the risk of 10% and 15% lung function decrements and used an exposure–response model that was more responsive to changes in exposure when the maximum daily 8-hr average (MDA8) O_3_ was > 40 ppb. In contrast, estimates of risk based on the application of results from epidemiology studies used linear, no-threshold concentration–response functions, so an incremental change in O_3_ affected total risk equally, regardless of the starting level of O_3_. As a result, the epidemiology-based risk estimates could be quite sensitive to the patterns of O_3_ responses to NO_x_ reductions, whereas the risk estimates based on controlled human exposure studies reflected the changes that occurred at the higher O_3_ concentrations.

Given the complex nature of O_3_ chemistry and emissions source mixes in a given urban area, photochemical grid models provide a credible tool for evaluating responsiveness to emissions changes and have been used extensively for O_3_ planning purposes in the past (Cai et al. 2011; Hogrefe et al. 2011; Kumar and Russell 1996; Simon et al. 2012). In the present study, we used a photochemical grid model applied in three different urban areas to illustrate temporal and spatial patterns of O_3_ and how those patterns may change as a result of reduced precursor emissions. Model-predicted changes in O_3_ were aggregated using several metrics to show how spatial heterogeneity depends on the O_3_ metric that is being analyzed and how the type of aggregation used can mute the spatial variability in O_3_ response to emissions changes.

## Methods

### City Selection And Monitoring Data

Three major urban areas (Philadelphia, Atlanta, and Chicago) were selected for evaluation based on the spatial coverage of the local ambient monitoring network and because they demonstrate different types of spatial O_3_ patterns and responses to emissions changes (U.S. EPA 2014a). In addition, the photochemical model performed well in these cities (U.S. EPA 2014a). For each urban area, all ambient monitoring sites within the combined statistical area (CSA) were included in the analysis as well as any additional monitoring sites within a 50-km buffer of the CSA boundary. Hourly average O_3_ data from these ambient monitoring sites for the years 2006–2008 were obtained from the U.S. EPA’s Air Quality System (AQS) database (http://www2.epa.gov/aqs).

### Ambient Data Adjustments to Estimate O_3_ Distributions under Alternate Emissions

We applied outputs from a series of photochemical air quality model simulations to estimate how hourly O_3_ could change under hypothetical scenarios of 50% and 75% reductions in U.S. anthropogenic NO_x_ emissions. Emissions projections from the U.S. EPA predict substantial reductions (45%) in U.S. anthropogenic NO_x_ between 2007 and 2020 (U.S. EPA 2012). VOC emissions are also expected to decline between 2007 and 2020 by a more modest 20% (U.S. EPA 2012). By evaluating O_3_ concentrations at 50% and 75% NO_x_ reductions, we were able to look at O_3_ responses for two scenarios of varying emissions. Although the primary focus of this study was on NO_x_ reduction scenarios, we also included a more limited evaluation of scenarios where both NO_x_ and VOC emissions were reduced by 50% and 75% to determine how cooccurring VOC reductions could change patterns of responses to NO_x_ reductions.

The methodology for adjusting observed O_3_ to scenarios of 50% and 75% NO_x_ reductions was adapted from the methods developed by Simon et al. (2013) and by the U.S. EPA (2014a). However, both of these studies targeted specific air quality goals rather than emissions reduction levels as were investigated here. The methods are described in detail in those documents and are summarized below.

We used the Community Multi-scale Air Quality (CMAQ) model v.4.7.1 instrumented with higher-order decoupled direct method (HDDM) capabilities to calculate O_3_ responses to emissions inputs. Details on the model setup and inputs are provided in the Supplemental Material (“Supplemental Methods”). Model predictions of MDA8 O_3_ were compared with monitored values in each of the three cities. Overall, the mean biases were 3.7 ppb, 2 ppb, and 1 ppb in Atlanta, Chicago, and Philadelphia, respectively. The Pearson correlations (*R*) were 0.82, 0.86, and 0.88, respectively. A seasonal breakout of these performance statistics is provided in Table S1. This model performance is within the range of what has been observed in state-of-the-science ozone modeling reported in recent studies (Simon et al. 2012) and is sufficiently accurate for the purpose of this analysis.

The CMAQ-HDDM system provided outputs of hourly O_3_ and hourly sensitivity coefficients at a 12 km × 12 km grid resolution across the contiguous United States. These sensitivities describe a nonlinear (quadratic) O_3_ response at a specified time and location to an across-the-board perturbation in NO_x_ emissions following Equation 1:


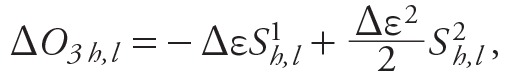
[1]

where Δ*O_3__h,l_* is the change in O_3_ at hour h and location l, Δε is the relative change in NO_x_ emissions (e.g., –0.2 represents a 20% reduction in NO_x_ emissions) and *S* ^^1^^ *_h,l_* and *S* ^^2^^ *_h,l_* represent the first- and second-order O_3_ sensitivity coefficients at hour *h* and location *l*.

Model simulations were performed for 7 months in 2007 (January and April–October), which provided O_3_ responses over a range of emissions and meteorological conditions and provided information on seasonal variations in the response. To apply the modeled sensitivity coefficients from 7 months to 3 years of ambient measurements (described above), we derived statistical relationships between the modeled sensitivity coefficients and the modeled hourly O_3_ concentrations using linear regression. Separate linear regressions were created for each monitor location at each hour of the day and for each season, resulting in a total of 96 (24 hr × 4 seasons) linear regressions for the first- and second-order sensitivity coefficients at each monitor location. The linear regression resulted in statistically significant relationships between ozone concentration and responsiveness to NO_x_ emissions reductions for most combinations of hours of the day, season, and monitoring location. Using these relationships, we could determine the first- and second-order sensitivity coefficients at O_3_ monitor locations for every hour of 2006–2008. Finally, we used Equation 1 to predict the change in measured ambient O_3_ concentrations for a set change in NO_x_ emissions at each monitor location.

**Figure 1:**
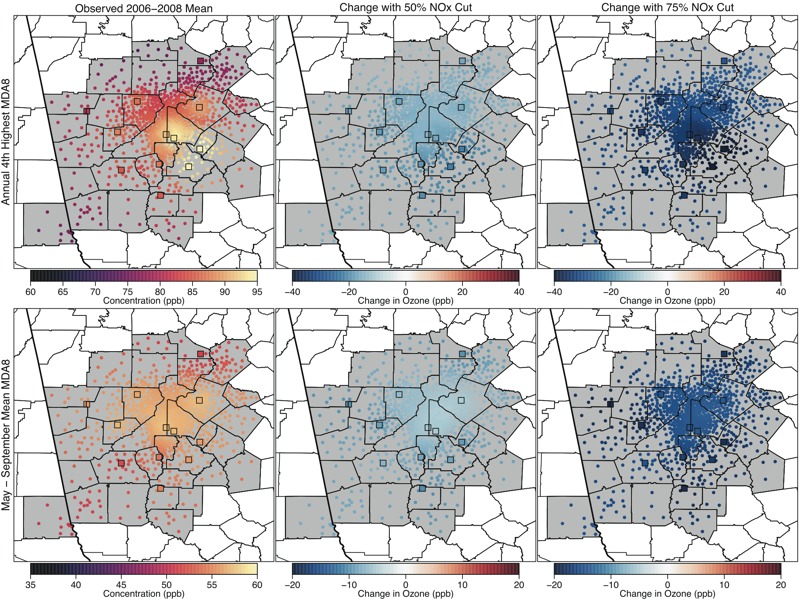
Maps showing the 2006–2008 average annual 4th-highest maximum daily 8-hr average ozone (MDA8 O_3_), the regulatory metric (parts per billion; top panels), and May–September mean MDA8 O_3_ (parts per billion; bottom panels) values in Atlanta for observed conditions (left panels), and predicted changes with 50% U.S. nitrogen oxide (NO_x_) emissions reductions (center panels) and 75% U.S. NO_x_ emissions reductions (right panels). Colored squares show locations of monitoring sites, and colored dots show interpolated values at census-tract centroids.

**Figure 2:**
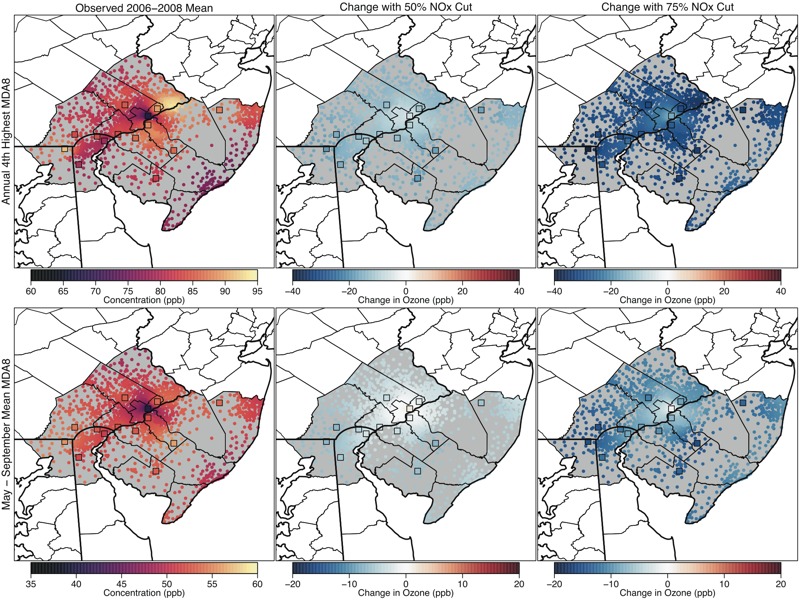
Maps showing the 2006–2008 average annual 4th-highest maximum daily 8-hr average ozone (MDA8 O_3_), the regulatory metric, (parts per billion; top panels) and May–September mean MDA8 O_3_ (parts per billion; bottom panels) values in Philadelphia for observed conditions (left panels), and predicted changes with 50% U.S. nitrogen oxide (NO_x_) emissions reductions (center panels) and 75% U.S. NO_x_ emissions reductions (right panels). Colored squares show locations of monitoring sites, and colored dots show interpolated values at census-tract centroids.

An additional complication is introduced when looking at large changes in NO_x_ emissions (e.g., 50% and 75%). Previous studies have reported that CMAQ-HDDM estimates of O_3_ changes are most accurate for emissions perturbations < 50% (Hakami et al. 2003). To address this issue, we ran the CMAQ-HDDM model at two distinct emissions levels (2007 emissions and 50% NO_x_ conditions) to derive emissions sensitivity coefficients that would occur under different emissions regimes. We then developed a two-step adjustment methodology in which sensitivity coefficients from each of the simulations were applied over the portion of emissions reductions for which they were most applicable (Simon et al. 2013; U.S. EPA 2014a). The exact point over the emissions reduction glide-path at which the sensitivity was switched differed for each city and was determined by minimizing the least square error between the adjusted O_3_ concentrations using the two-step approach and actual modeled concentrations from “brute force” NO_x_ cut simulations. Analyses presented by the U.S. EPA (2014a) showed that this two-step approach could replicate O_3_ changes for 50% emissions reductions with mean bias between –0.2 and –0.9 ppb and mean absolute error equal to 1 ppb for the three cities evaluated here.

### Spatial Interpolation

To better examine spatial patterns in O_3_ concentrations and population exposure, we used the Voronoi neighbor averaging (VNA) (Gold et al. 1997; Chen et al. 2004) method to create spatial fields of the hourly O_3_ in each urban area for the observed, 50% NO_x_ cut, and 75% NO_x_ cut scenarios. We used VNA to interpolate the observed or adjusted hourly concentration data using an inverse distance squared weighting from monitored locations to each census tract centroid within the CSA boundaries of each urban area. The resulting spatial fields provided temporally complete estimates of observed and adjusted hourly O_3_ concentrations at refined spatial resolution within each urban area. Previous cross-validation tests showed that this interpolation method provided good performance at urban scales (U.S. EPA 2014a).

## Results

### Predicted Spatial Changes

**Figure 3:**
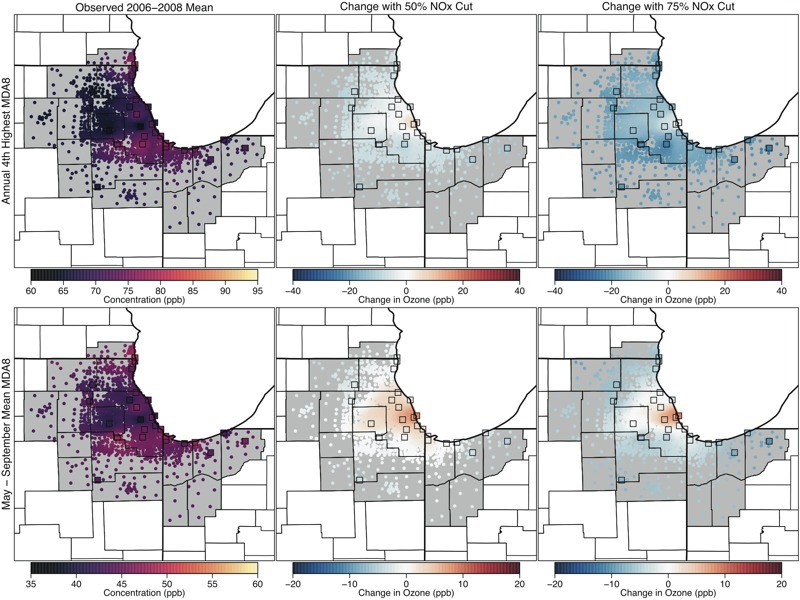
Maps showing the 2006–2008 average annual 4th-highest maximum daily 8-hr average ozone (MDA8 O_3_), the regulatory metric, (parts per billion; top panels) and May–September mean MDA8 O_3_ (parts per billion; bottom panels) values in Chicago for observed conditions (left panels), and predicted changes with 50% U.S. nitrogen oxide (NO_x_) emissions reductions (center panels) and 75% U.S. NO_x_ emissions reductions (right panels). Colored squares show locations of monitoring sites, and colored dots show interpolated values at census-tract centroids.

We focused on two MDA8 O_3_ metrics for our assessment: the annual 4th-highest MDA8 value averaged over 3 consecutive years, which corresponds to the form and averaging time of the current O_3_ NAAQS and represents O_3_ concentrations on the highest O_3_ days (also known as the “design value” and hereafter referred to as the regulatory metric), and the May–September mean of the MDA8 (i.e., the seasonal mean metric), which generally tracks well with estimates of epidemiology-based O_3_ health risk (U.S. EPA 2014a). Maps of VNA surfaces showing spatial patterns in these metrics for Atlanta, Philadelphia, and Chicago are presented in Figures 1–3. The left panels show the values based on measured O_3_ concentrations, and the center and right panels show the changes in these observed values under the 50% and 75% NO_x_ cut scenarios, respectively.

For the observed scenario, Atlanta had fairly uniform O_3_ concentrations (particularly for the seasonal average), whereas Philadelphia and Chicago had sharp spatial gradients owing to local O_3_ suppression near NO emissions sources in the urban core and subsequent O_3_ formation downwind as described in the introduction. When considering the predicted changes in the two metrics under the 50% and 75% NO_x_ cut scenarios, each city displayed a different spatial pattern. In Atlanta, large NO_x_ reductions were predicted to result in fairly uniform decreases in both O_3_ metrics across the CSA, although the decreases were slightly less pronounced in the urban core. In Philadelphia, the regulatory metric was predicted to decrease everywhere, whereas the seasonal mean metric was predicted to increase slightly in a small area of the urban core under 50% NO_x_ reduction and to decrease in the rest of the CSA. Notably, the increases in the seasonal mean tended to occur in locations with lower observed values, whereas decreases tended to occur in locations with higher observed values, resulting in a more uniform spatial gradient. The 75% NO_x_ cut scenario predicted decreases in both the regulatory metric and the seasonal mean metric throughout the entire Philadelphia CSA, with decreases being more pronounced in outlying areas. Finally, Chicago displayed a third type of response pattern. For the 50% NO_x_ reduction scenario, the regulatory metric in Chicago was predicted to increase slightly in the urban core and near Lake Michigan, whereas it decreased in surrounding locations. For the 75% reduction scenario, the regulatory metric showed substantial decreases, whereas the seasonal mean concentrations continued to increase in the urban core. The spatial extent of the area where the seasonal mean was predicted to increase was larger than the spatial extent of the area where regulatory-metric O_3_ was predicted to increase. Figures for spatial patterns in two additional cities (Denver and Sacramento) are provided in Figures S1 and S2. The patterns in Denver resembled those seen in Philadelphia, whereas the patterns in Sacramento most resembled those in Atlanta.

**Figure 4:**
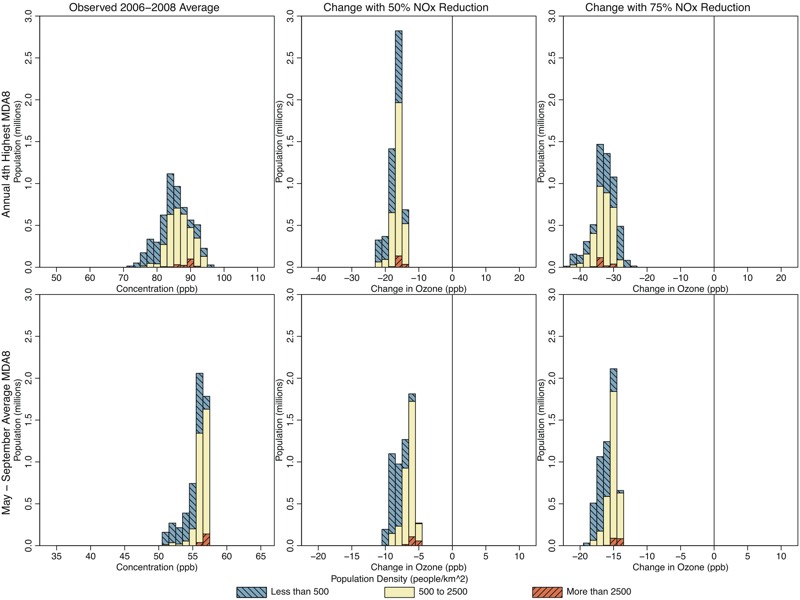
Histograms showing population living in Atlanta locations with various 2006–2008 average 4th-highest maximum daily 8-hr average ozone (MDA8 O_3_) (top panels; parts per billion) and May–September mean MDA8 O_3_ (bottom panels; parts per billion), for observed conditions (left panels), and predicted changes resulting from 50% U.S. nitrogen oxide (NO_x_) emissions reductions (center panels) and 75% U.S. NO_x_ reductions (right panels). Colors show the breakdown of each histogram by population density.

**Figure 5:**
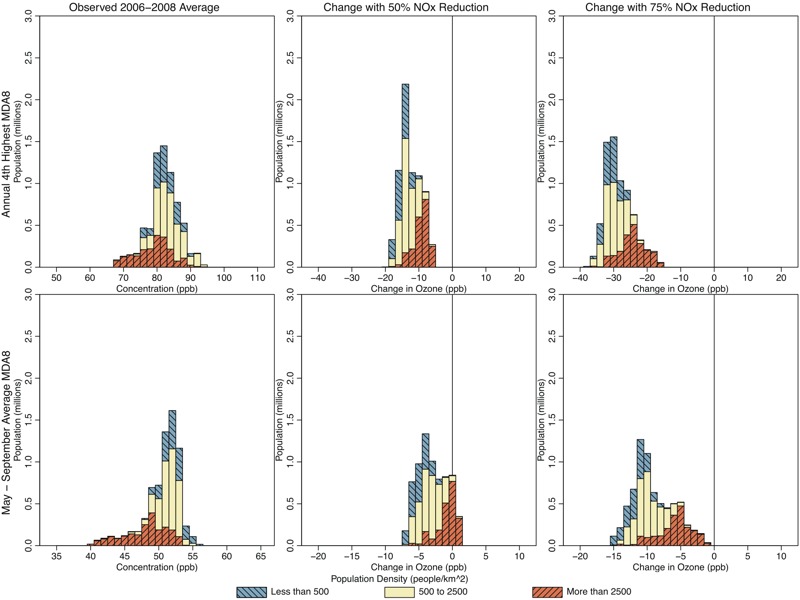
Histograms showing population living in Philadelphia locations with various 2006–2008 average 4th-highest maximum daily 8-hr average ozone (MDA8 O_3_) (top panels; parts per billion) and May–September mean MDA8 O_3_ (bottom panels; parts per billion), for observed conditions (left panels), and predicted changes resulting from 50% U.S. nitrogen oxide (NO_x_) emissions reductions (center panels) and 75% U.S. NO_x_ reductions (right panels). Colors show the breakdown of each histogram by population density.

**Figure 6:**
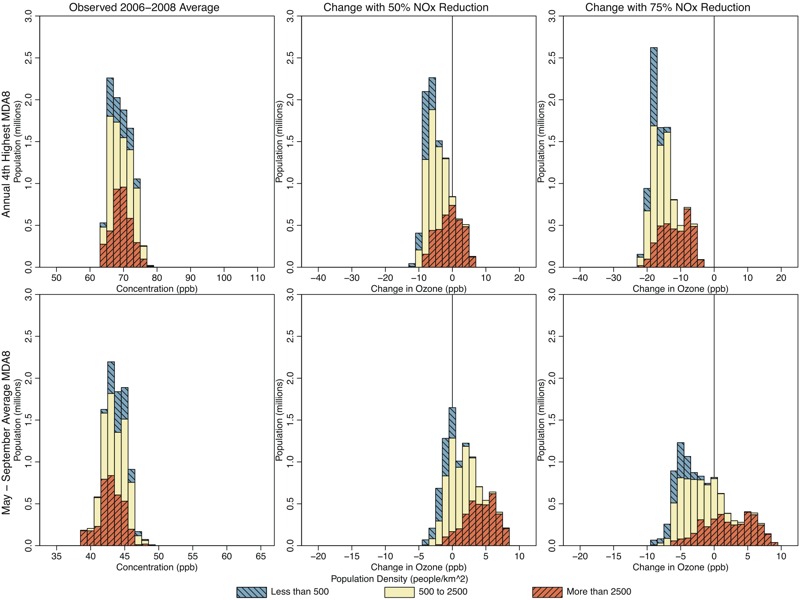
Histograms showing population living in Chicago locations with various 2006–2008 average 4th-highest maximum daily 8-hr average ozone (MDA8 O_3_) (top panels; parts per billion) and May–September mean MDA8 O_3_ (bottom panels; parts per billion), for observed conditions (left panels), and predicted changes resulting from 50% U.S. nitrogen oxide (NO_x_) emissions reductions (center panels) and 75% U.S. NO_x_ emissions reductions (right panels). Colors show the breakdown of each histogram by population density.

**Figure 7:**
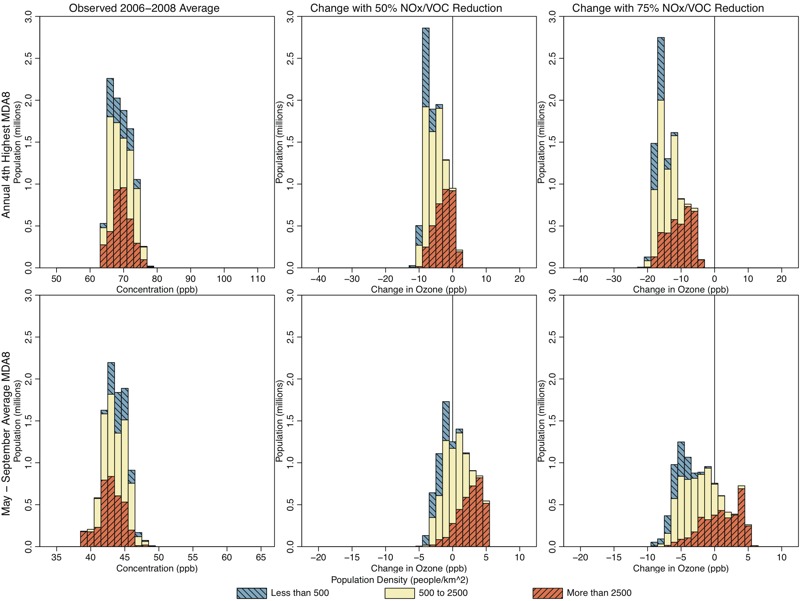
Histograms showing population living in Chicago locations with various 2006–2008 average 4th-highest maximum daily 8-hr average ozone (MDA8 O_3_) (top panels; parts per billion) and May–September mean MDA8 O_3_ (bottom panels; parts per billion), for observed conditions (left panels), and predicted changes resulting from 50% U.S. nitrogen oxide/volatile organic compound (NO_x_/VOC) emissions reductions (center panels) and 75% U.S. NO_x_/VOC emissions reductions (right panels). Colors show the breakdown of each histogram by population density.

Figures 4–7 show the populations (2010 census, http://factfinder.census.gov/faces/nav/jsf/pages/index.xhtml) of locations predicted to have increasing and decreasing O_3_ using the regulatory and seasonal mean metrics in each of the three cities. In general, the least densely populated locations had the highest O_3_ and the largest decreases in O_3_. Conversely, the most densely populated locations had the lowest O_3_ and the smallest decreases, or in some cases increases, in O_3_. Several specific features can be observed in these histograms. First, in Atlanta and Philadelphia, the entire population lived in locations where the regulatory metric was predicted to decrease. In Chicago, although a small area near the urban core was predicted to show increases in the regulatory metric (50% NO_x_ reduction scenario only), the vast majority of the population lived in locations where the regulatory metric was predicted to decrease. Second, consistent with results shown in Figure 1, seasonal mean O_3_ was predicted to decrease in all locations of Atlanta. In Philadelphia, there is a small area that was predicted to have increases in seasonal mean O_3_ in the 50% NO_x_ reduction scenario but not in the 75% NO_x_ reduction scenario. Consequently, a small fraction of the Philadelphia population lived in locations of low but somewhat increasing seasonal mean O_3_ in the 50% NO_x_ reduction scenario. The net result of these changes in both Atlanta and Philadelphia is that the O_3_ distribution was compressed as well as shifted toward lower concentrations for both metrics and in both the 50% and 75% NO_x_ cut scenarios (see Figures S3 and S4). Finally, compared with the other two cities, Chicago was predicted to have a larger area of increasing seasonal mean O_3_, and consequently, a larger fraction of Chicago residents (55% and 26% for the 50% and 75% NO_x_ reduction scenarios, respectively) living in those locations (see Figure S5). Denver and Sacramento results are shown in Figures S6–S9.

Previous work has shown that VOC emissions reductions can result in lower ambient O_3_ levels (Sillman 1999) and that changes in ambient VOC concentrations can affect how O_3_ responds to NO_x_ emissions reductions (Chameides et al. 1988). Of the cities we analyzed, previous analysis suggests that anthropogenic VOC emissions reductions will result in the most notable estimated O_3_ reductions for the Chicago area (U.S. EPA 2014a). We therefore conducted additional simulations where both anthropogenic VOCs and NO_x_ were reduced in Chicago. These simulations resulted in lower regulatory and seasonal metrics than simulations conducted with NO_x_ emissions reductions alone (Figure 6 and 7). However, the combined levels of NO_x_ and VOC emissions reductions applied here still resulted in some increases in low O_3_ in the urban core, indicating that the inclusion of VOC reductions does not eliminate the need to understand how heterogeneous O_3_ responses to emission reductions can affect exposure.

### Predicted Temporal Changes

**Figure 8:**
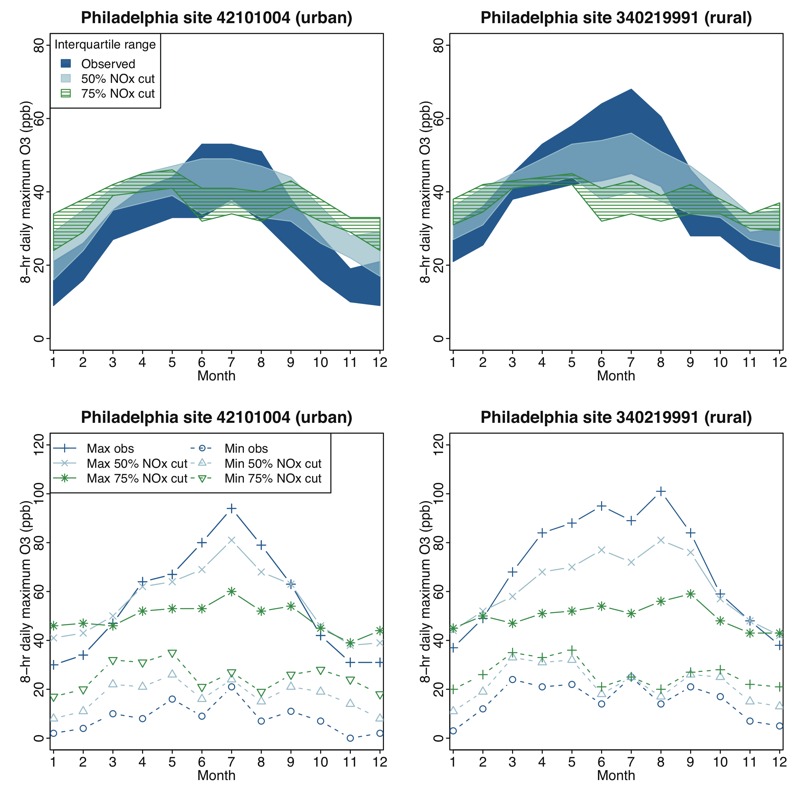
Distribution of 8-hr daily maximum ozone (O_3_) concentrations in Philadelphia by month at an urban (left panels) and a rural (right panels) monitoring site. Top plots show the interquartile range (25th to 75th percentile values) across all days in each month. Bottom plots show minimum and maximum values across all days in each month.

Figure 8 and Figure S10 show the seasonal pattern in O_3_ concentrations at an urban monitor in Philadelphia and at a rural downwind monitor over all days with measured values in 2006–2008. As expected, the highest concentrations were observed in the summer months, with lower O_3_ observed in the fall and winter. This pattern shifted in the 50% and 75% NO_x_ reduction scenarios. First, the monthly maximum concentrations generally decreased while the monthly minimum concentrations increased, leading to a compression of the O_3_ distribution. Decreases in monthly maximum values were largest during the summer, and increases in monthly minimum values were largest in the winter. Second, midrange O_3_ values (see top panels of Figure 8) were generally predicted to decrease during the summer and to increase in the winter months. The behavior in spring and fall differed at the two locations, with increases in both seasons at the urban site but decreases in spring at the rural site. These differences led to a flattening of the seasonal O_3_ pattern for both extreme and midrange O_3_ values. Finally, a shift in the seasonal peak for midrange O_3_ concentrations was evident in the 75% NO_x_ reduction scenario (compare the hatched green and dark blue ribbons in the top panels of Figure 8). Whereas the observed midrange concentrations reached their peak in the summer (June, July, August), the midrange concentrations in the 75% NO_x_ reduction scenario were predicted to be highest in the spring months (March, April, May), with a secondary peak in the fall. This shift was not seen for monthly maximum values. The shifting O_3_ seasonal patterns are similar to results reported by Clifton et al. (2014), who used global modeling to predict O_3_ increases in winter and decreases in summer on a regional scale. This general phenomenon is corroborated by recent studies analyzing observed O_3_ trends corresponding to decreasing NO_x_ emissions over the past decade, which showed that winter O_3_ concentrations have been increasing, whereas summer concentrations have been decreasing (Jhun et al. 2015; Simon et al. 2015).

Changes in diurnal patterns at these two monitoring sites are shown in Figure S11. Nighttime concentrations were predicted to increase with decreasing NO_x_, whereas daytime concentrations were predicted to decrease. Similar to changes in the seasonal pattern, these changes generally led to a flattening out of the diurnal pattern. Increases were more pronounced at the urban monitor, where concentrations were low, than at the rural monitor, where concentrations were high.

Temporal plots for the other four cities showed generally similar patterns to those observed in Philadelphia and can be found in Figures S12–S19.

## Discussion

### Limitations and Uncertainties

We note several limitations and uncertainties. First, we looked at two scenarios of across-the-board reductions in U.S. anthropogenic NO_x_ (and VOC) emissions as case studies to demonstrate how changing emissions could affect spatial and temporal patterns of O_3_. Our modeling explored broad regional reductions in NO_x_ (and VOC) emissions, and we have not evaluated whether more spatially focused reductions could be equally effective in reducing peak concentrations while avoiding increases in O_3_ in urban core areas. Future research may explore strategies for mitigating NO_x_-related increases in O_3_. In addition, different sources are likely to reduce their emissions at different rates. This analysis demonstrated how spatial and temporal O_3_ patterns and resulting exposure may change with emissions reductions but did not attempt to predict future emissions levels or the associated effects on O_3_. Second, we applied modeled O_3_ responses from 12-km–resolution regional model simulations to ambient measurements at O_3_ monitors. Although 12-km–resolution modeling has generally been shown to capture most features of O_3_ response, the variability in predicted O_3_ responses can be somewhat muted compared with that found in modeling performed at finer grid resolutions (Cohan et al. 2006). Third, we note that in Philadelphia, the increasing seasonal mean values were only predicted at a single monitor, and in Chicago, increases in the regulatory metric were only predicted at two monitors. In both cases, there was some uncertainty in the VNA interpolations showing the spatial extent of those increases. Finally, this analysis used 7 months of modeling data to represent O_3_ responses across 3 years. The modeling simulated at least 1 representative month for each season, allowing us to create separate regressions for each site, hour of the day, and season, which enabled us to capture seasonal variability in the response of O_3_ to emissions changes. In addition, both meteorology (e.g., ozone-conducive conditions) and emissions in 2006 and 2008 may have varied from those in the 2007 modeling year. Therefore, the application of regressions based on conditions from 7 months in 2007 to 2006–2008 observations is another source of uncertainty.

### Implications for Future Epidemiology Studies and Risk Assessments

The results from this analysis show substantial heterogeneity in O_3_ responses within CSAs. These findings suggest that the composite monitors used in many health studies may mask potentially important aspects of the impact of NO_x_ changes on O_3_ distributions (changes to high vs. low O_3_ concentrations, spatial variability, and temporal patterns) and how they relate to total population-weighted exposure.

First, a key observation from our analysis is that for all three areas, O_3_ concentrations using the regulatory metric were reduced at locations with the highest concentrations (which are generally the monitors that are targeted when selecting NO_x_ or VOC emissions controls). Additionally, monthly maximum O_3_ was decreased at both urban and rural sites under the 50% and 75% NO_x_ cut scenarios (Figure 8). As noted earlier, some measures of risk, such as the lung function responses derived from controlled human exposure studies, are most sensitive to these reductions in high concentrations, and thus, these risk metrics will show improvement with decreases in NO_x_. In contrast, the epidemiology-based risks that are currently equally responsive to changes at low and high concentrations are more ambiguous in response to NO_x_ decreases and ultimately depend on the direction and magnitude of the O_3_ change in places with the highest population densities. Because increases in O_3_ almost always occur at low O_3_ concentrations, it is increasingly important for health studies to evaluate whether the shape of the concentration–response relationship changes at these lower levels.

Second, spatial gradients of ambient O_3_ are evident in each of the urban areas shown, and there is both spatial and temporal heterogeneity in the response of ambient O_3_ to NO_x_ decreases. This heterogeneity enhances the importance of understanding where and when people spend their days when linking ambient monitoring data to health outcomes. The U.S. EPA (2014a) found that when population exposures to O_3_ are modeled using time–activity information, the highest exposures to O_3_ occur for children spending a large amount of time outdoors during high-O_3_ periods and for adult outdoor workers in high-O_3_ areas. These findings suggest that epidemiology studies could be improved and that exposure misclassification could be reduced by providing population exposure surrogates that represent time–activity weighted patterns of O_3_ exposure instead of using simpler spatial averages or single-point measures that assume individuals spend all of their time at a residential location. In addition, time–activity weighted measures of population exposure to O_3_ will be better able to capture the changes in spatial and temporal patterns of O_3_ that can result from reductions in O_3_ precursor emissions to attain the NAAQS.

Third, the model predictions that increases in O_3_ are more prevalent in cooler months and that decreases are more prevalent in warmer months may be important if activities that lead to increased exposure (such as time spent outdoors) are practiced less frequently in winter than in summer. Some research suggests that health impacts of O_3_ may be lower in cooler months than in warmer months (Medina-Ramón 2006; Stieb et al. 2009; Strickland et al. 2010). To the extent that O_3_ health impacts vary by season, a fuller characterization of exposure patterns may improve understanding of the implications of the predicted changes in seasonal O_3_ patterns.

## Conclusion

The net impact of emissions reductions on health over an entire population will depend on the balance of O_3_ changes across high- and low-population-density locations and across the entire O_3_ season as well as on the shape of the concentration–response relationship at different concentrations. Our analysis highlights the potential impacts of NO_x_ emissions reductions on spatial and temporal patterns of O_3_. Extensions of this work that elucidate the relationships between population time–activity patterns (as well as potentially affected communities) and these changes in the spatial and temporal patterns of O_3_ may allow regulators to consider modifications to NO_x_ and VOC reduction plans (e.g., to avoid increases in O_3_ at times and locations when they are most likely to result in negative health outcomes).

## Supplemental Material

(5 MB) PDF
