# Supplementary material for: Assessing Temporal and Spatial Patterns of Observed and Predicted Ozone in Multiple Urban Areas
Source: Environ Health Perspect. 2016 May 6;124(9):1443–52. doi: 10.1289/EHP190 (PMC5010397; doi:10.1289/EHP190)
Supplement: (5 MB) PDF [file EHP190.s001.acco.pdf]

Environ Health Perspect
DOI: 10.1289/EHP190

Note to readers with disabilities: *EHP* strives to ensure that all journal content is accessible to all readers. However, some figures and Supplemental Material published in *EHP* articles may not conform to 508 standards due to the complexity of the information being presented. If you need assistance accessing journal content, please contact ehp508@niehs.nih.gov. Our staff will work with you to assess and meet your accessibility needs within 3 working days.

Supplemental Material

Assessing Temporal and Spatial Patterns of Observed and Predicted Ozone in Multiple Urban Areas

Heather Simon, Benjamin Wells, Kirk R. Baker, and Bryan Hubbell

Contents

Supplemental Methods

Table S1. Model performance statistics for MDA8 ozone in Atlanta, Chicago, and Philadelphia

Supplemental Results

Figure S1. Maps showing the 2006-2008 average annual 4th highest MDA8 O_3, the regulatory metric, (ppb, top panels) and May-September mean MDA8 O_3 (ppb, bottom panels) values in Denver for observed conditions (left panels), and predicted changes with 50% U.S. NO_x emissions reductions (center panels) and 75% U.S. NO_x emissions reductions (right panels). Black boxes show locations of monitoring sites while colored dots show interpolated values at census tract centroids

Figure S2. Maps showing the 2006-2008 average annual 4th highest MDA8 O_3, the regulatory metric, (ppb, top panels) and May-September mean MDA8 O_3 (ppb, bottom panels) values in Sacramento for observed conditions (left panels), and predicted changes with 50% U.S. NO_x emissions reductions (center panels) and 75% U.S. NO_x emissions reductions (right panels). Black boxes show locations of monitoring sites while colored dots show interpolated values at census tract centroids

DOI: 10.1289/EHP190

Figure S3. Histograms showing population living in Atlanta locations with various 2006-2008 average 4th highest MDA8 O3 (top panels, ppb) and May – September mean MDA8 O3 (bottom panels, ppb), for observed conditions (left panels), and predicted resulting conditions resulting from 50% U.S. NOx emissions reductions (center panels) and 75% U.S. NOx reductions (right panels). Colors show the breakdown of each histogram by population density.

Figure S4. Histograms showing population living in Philadelphia locations with various 2006-2008 average 4th highest MDA8 O3 (top panels, ppb) and May – September mean MDA8 O3 (bottom panels, ppb), for observed conditions (left panels), and predicted resulting conditions resulting from 50% U.S. NOx emissions reductions (center panels) and 75% U.S. NOx reductions (right panels). Colors show the breakdown of each histogram by population density.

Figure S5. Histograms showing population living in Chicago locations with various 2006-2008 average 4th highest MDA8 O3 (top panels, ppb) and May – September mean MDA8 O3 (bottom panels, ppb), for observed conditions (left panels), and predicted resulting conditions resulting from 50% U.S. NOx emissions reductions (center panels) and 75% U.S. NOx reductions (right panels). Colors show the breakdown of each histogram by population density.

Figure S6. Histograms showing population living in Denver locations with various 2006-2008 average 4th highest MDA8 O3 (top panels, ppb) and May – September mean MDA8 O3 (bottom panels, ppb), for observed conditions (left panels), and predicted changes resulting from 50% U.S. NOx emissions reductions (center panels) and 75% U.S. NOx reductions (right panels). Colors show the breakdown of each histogram by population density

Figure S7. Histograms showing population living in Denver locations with various 2006-2008 average 4th highest MDA8 O3 (top panels, ppb) and May – September mean MDA8 O3 (bottom panels, ppb), for observed conditions (left panels), and predicted resulting conditions resulting from 50% U.S. NOx emissions reductions (center panels) and 75% U.S. NOx reductions (right panels). Colors show the breakdown of each histogram by population density.

Figure S8. Histograms showing population living in Sacramento locations with various 2006-2008 average 4th highest MDA8 O3 (top panels, ppb) and May – September mean MDA8 O3 (bottom panels, ppb), for observed conditions (left panels), and predicted changes resulting from 50% U.S. NOx emissions reductions (center panels) and 75% U.S. NOx reductions (right panels). Colors show the breakdown of each histogram by population density

Figure S9. Histograms showing population living in Sacramento locations with various 2006-2008 average 4th highest MDA8 O3 (top panels, ppb) and May – September mean MDA8 O3 (bottom panels, ppb), for observed conditions (left panels), and predicted resulting conditions resulting from 50% U.S. NOx emissions reductions (center panels) and 75% U.S. NOx reductions (right panels). Colors show the breakdown of each histogram by population density.

Figure S10. Distribution of 8-hr daily maximum O3 concentrations in Philadelphia by month at an urban and a rural monitoring site. Gray boxes show observed distribution. Yellow and blue boxes show predicted distribution after 50% and 75% reductions in US anthropogenic NOx emissions respectively. Horizontal bars show medial values, boxes outline the interquartile range, whiskers outline 1.5 times the interquartile range, and dots show outlier values

Figure S11. Distribution of hourly O3 concentrations in Philadelphia by hour of the day at an urban and a rural monitoring site. Gray boxes show observed distribution. Yellow and blue boxes show predicted distribution after 50% and 75% reductions in US anthropogenic NOx emissions respectively. Horizontal bars show medial values, boxes outline the interquartile range, whiskers outline 1.5 times the interquartile range, and dots show outlier values

Figure S12. Distribution of 8-hr daily maximum O3 concentrations in Chicago by month at an urban and a rural monitoring site. Gray boxes show observed distribution. Yellow and blue boxes show predicted distribution after 50% and 75% reductions in US anthropogenic NOx emissions respectively. Horizontal bars show medial values, boxes outline the interquartile range, whiskers outline 1.5 times the interquartile range, and dots show outlier values

Figure S13. Distribution of hourly O3 concentrations in Chicago by hour of the day at an urban and a rural monitoring site. Gray boxes show observed distribution. Yellow and blue boxes show predicted distribution after 50% and 75% reductions in US anthropogenic NOx emissions respectively. Horizontal bars show medial values, boxes outline the interquartile range, whiskers outline 1.5 times the interquartile range, and dots show outlier values

Figure S14. Distribution of 8-hr daily maximum O3 concentrations in Atlanta by month at an urban and a rural monitoring site. Gray boxes show observed distribution. Yellow and blue boxes show predicted distribution after 50% and 75% reductions in US anthropogenic NOx emissions respectively. Horizontal bars show medial values, boxes outline the interquartile range, whiskers outline 1.5 times the interquartile range, and dots show outlier values

Figure S15. Distribution of hourly O3 concentrations in Atlanta by hour of the day at an urban and a rural monitoring site. Gray boxes show observed distribution. Yellow and blue boxes show predicted distribution after 50% and 75% reductions in US anthropogenic NOx emissions respectively. Horizontal bars show medial values, boxes outline the interquartile range, whiskers outline 1.5 times the interquartile range, and dots show outlier values

Figure S16. Distribution of 8-hr daily maximum O3 concentrations in Denver by month at an urban and a suburban monitoring site. Gray boxes show observed distribution. Yellow and blue boxes show predicted distribution after 50% and 75% reductions in US anthropogenic NOx emissions respectively. Horizontal bars show medial values, boxes outline the interquartile range, whiskers outline 1.5 times the interquartile range, and dots show outlier values

Figure S17. Distribution of hourly O3 concentrations in Denver by hour of the day at an urban and a suburban monitoring site. Gray boxes show observed distribution. Yellow and blue boxes show predicted distribution after 50% and 75% reductions in US anthropogenic NOx emissions respectively. Horizontal bars show medial values, boxes outline the interquartile range, whiskers outline 1.5 times the interquartile range, and dots show outlier values

Figure S18. Distribution of 8-hr daily maximum O3 concentrations in Sacramento by month at an urban and a rural monitoring site. Gray boxes show observed distribution. Yellow and blue boxes show predicted distribution after 50% and 75% reductions in US anthropogenic NOx emissions respectively. Horizontal bars show medial values, boxes outline the interquartile range, whiskers outline 1.5 times the interquartile range, and dots show outlier values

Figure S19. Distribution of hourly O3 concentrations in Sacramento by hour of the day at an urban and a rural monitoring site. Gray boxes show observed distribution. Yellow and blue boxes show predicted distribution after 50% and 75% reductions in US anthropogenic NOx emissions respectively. Horizontal bars show medial values, boxes outline the interquartile range, whiskers outline 1.5 times the interquartile range, and dots show outlier values

Supplemental References

Supplemental Methods

Set-up, inputs, and performance evaluation results for the air quality modeling used in this analysis are described in more detail in EPA, 2014. Below is a brief summary.

Photochemical model simulations were performed using CMAQv4.7.1 with HDDM for ozone (O_3) (www.cmaq-model.org) (Foley et al. 2010). CMAQ was run using the carbon bond 2005 (CB05) gas-phase chemical mechanism (Gery et al. 1989; Yarwood et al. 2005). The modeling domain covered the 48 contiguous states and portions of southern Canada and Northern Mexico with a 12 x 12 km resolution. The domain extended upward to 17,600 meters, or 50 millibars (mb), using 24 vertical layers. Model simulations were run for January and April-October of 2007. The simulations included 10 day "ramp-up" periods from December 22-31, 2006, and from March 22-31 2007, to minimize the effects of initial conditions. The ramp-up days were not utilized in the analysis.

CMAQ model simulations require inputs of meteorological fields, emissions, and initial and boundary conditions. The gridded meteorological data for the entire year of 2007 at the 12 km continental United States scale domain was derived from version 3.1 of the Weather Research and Forecasting Model (WRF), Advanced Research WRF (ARW) core (Shamarock et al. 2008). The emissions data used are based on the 2007 Version 5 emissions modeling platform developed for the Particulate Matter (PM) NAAQS rule (U.S. EPA 2012a, U.S. EPA 2012b) with some minor updates. Inputs were included for emissions from U.S. anthropogenic sources (electric generating utilities, other point sources, area sources, onroad vehicles, and nonroad mobile sources), wildfires and prescribed burns, biogenic sources (estimated using the Biogenic Emissions Inventory System version 3.14 (BEISv3.14)) and

Canadian and Mexican emissions based on a 2006 and a 2008 inventory respectively. The lateral boundary concentrations for the 12km US2 domain are provided by a three-dimensional global atmospheric chemistry model, the GEOS-CHEM (Yantosca 2004) model (standard version 8-03-02 with version 8-02-03 chemistry). A 2007 GEOS-CHEM simulation was run with a grid resolution of 2.0 degree x 2.5 degree (latitude-longitude) and 46 vertical layers up to 0.01 hPa. The predictions were processed using the GEOS-2-CMAQ tool (Akhtar et al. 2012, Henderson et al. 2013) and used to provide one-way dynamic boundary conditions at one-hour intervals. Initial conditions were extracted from a slightly older model simulation using GEOS-CHEM version 8-02-03. The model simulation from which the initial conditions were extracted was also run with a grid resolution of 2.0 of 2.0 degree x 2.5 degree (latitude-longitude) and 47 vertical layers.

A model performance evaluation was conducted by comparing model-predicted MDA8 ozone concentrations to observations at ambient ozone monitors in the three cities discussed in the main paper. Observations and model predictions were matched in space and time for 11 monitors in Atlanta, 24 monitors in Chicago and 16 monitors in Philadelphia. Figure S-1 shows mean bias (ppb), normalized mean bias (%) and the Pearson correlation (R) segregated by season and aggregated across the entire year for each city. A model performance evaluation that includes time series plots and maps of model bias is available in US EPA (2014).

Table S1. Model performance statistics for MDA8 ozone in Atlanta, Chicago, and Philadelphia

	Atlanta			Chicago			Philadelphia		
	MB (ppb)	NMB (%)	**R**	**MB (ppb)**	**NMB (%)**	**R**	**MB (ppb)**	**NMB (%)**	**R**
winter	N/A	N/A	N/A	-3.9	-17.5	0.73	-3.6	-14.6	0.8
spring	-0.5	-1	0.75	-0.1	-0.2	0.82	-1.8	-3.8	0.83
summer	7	11.9	0.81	6.8	13.9	0.72	4.7	8.5	0.81
fall	5.3	12.1	0.85	1.3	3.8	0.89	2.4	6.4	0.83
all seasons	3.7	7	0.82	2	5	0.86	1	2.3	0.88

DOI: 10.1289/EHP190

Supplemental Results

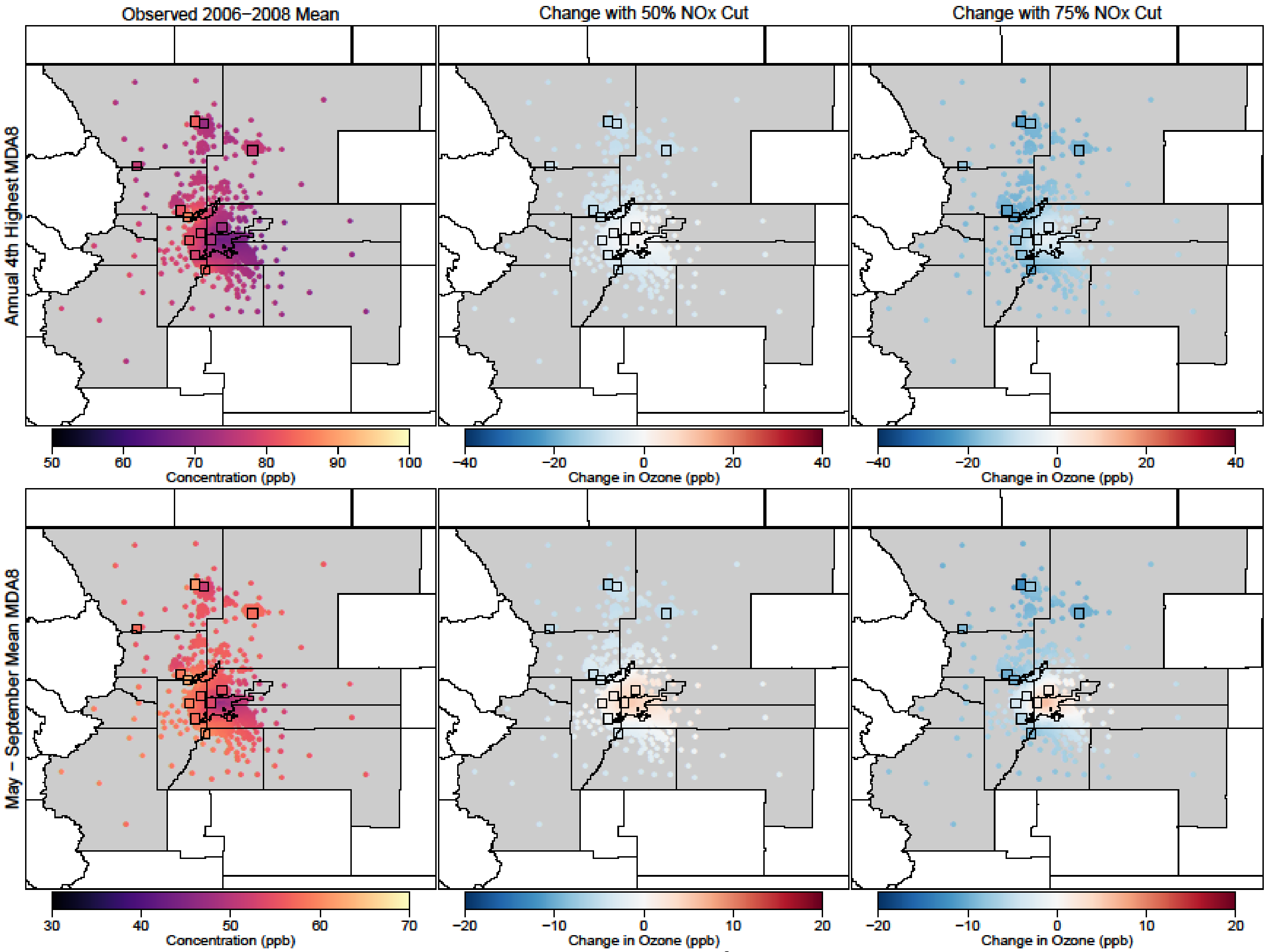

Figure S1. Maps showing the 2006-2008 average annual 4th highest MDA8 O_3, the regulatory metric, (ppb, top panels) and May-September mean MDA8 O_3 (ppb, bottom panels) values in Denver for observed conditions (left panels), and predicted changes with 50% U.S. NO_x emissions reductions (center panels) and 75% U.S. NO_x emissions reductions (right panels). Black boxes show locations of monitoring sites while colored dots show interpolated values at census tract centroids. Note scale is different from scale used in figures shown in main paper.

DOI: 10.1289/EHP190

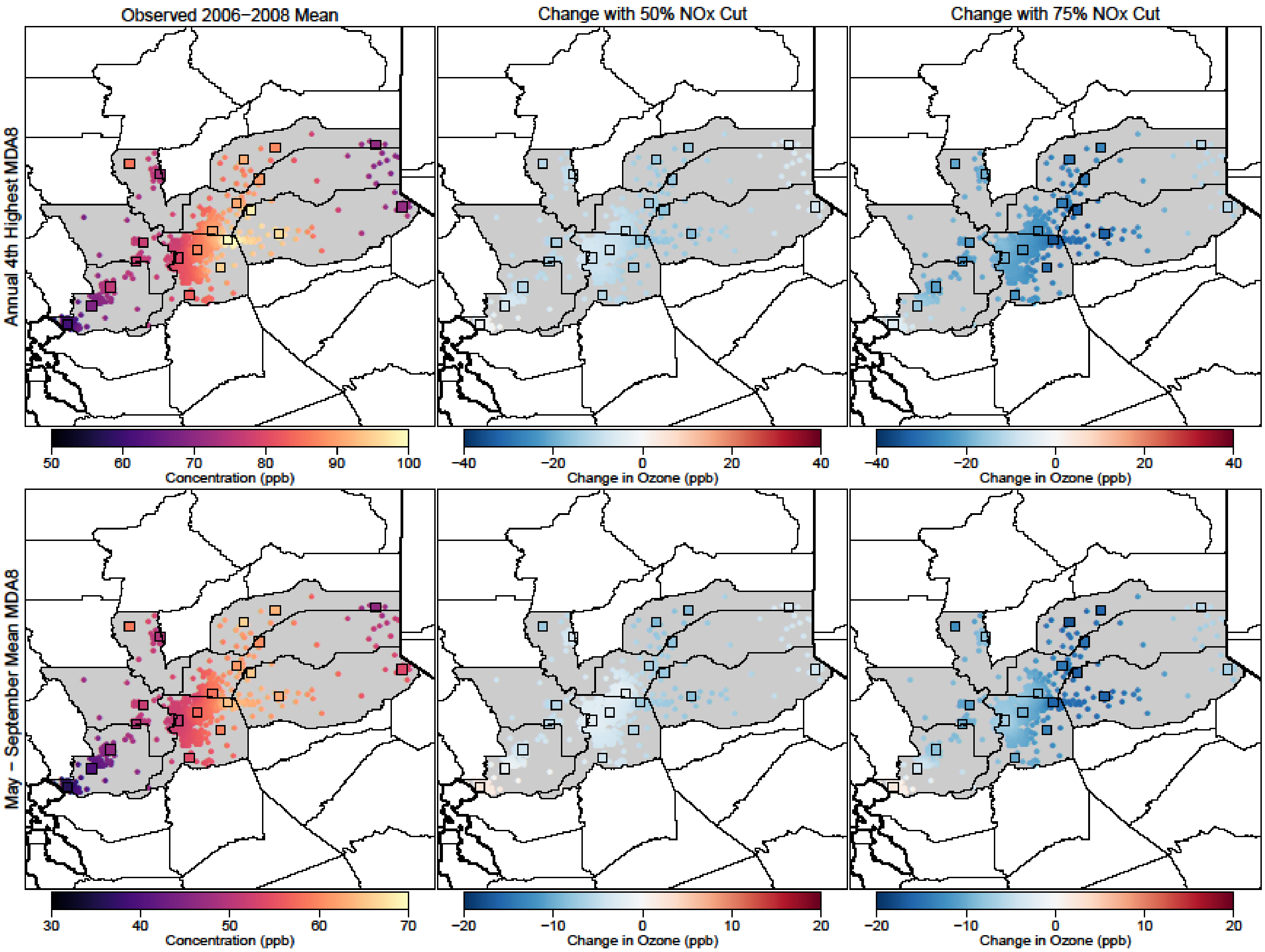

Figure S2. Maps showing the 2006-2008 average annual 4th highest MDA8 O_3, the regulatory metric, (ppb, top panels) and May-September mean MDA8 O_3 (ppb, bottom panels) values in Sacramento for observed conditions (left panels), and predicted changes with 50% U.S. NO_x emissions reductions (center panels) and 75% U.S. NO_x emissions reductions (right panels). Black boxes show locations of monitoring sites while colored dots show interpolated values at census tract centroids. Note scale is different from scale used in figures shown in main paper.

Environ Health Perspect
DOI: 10.1289/EHP190

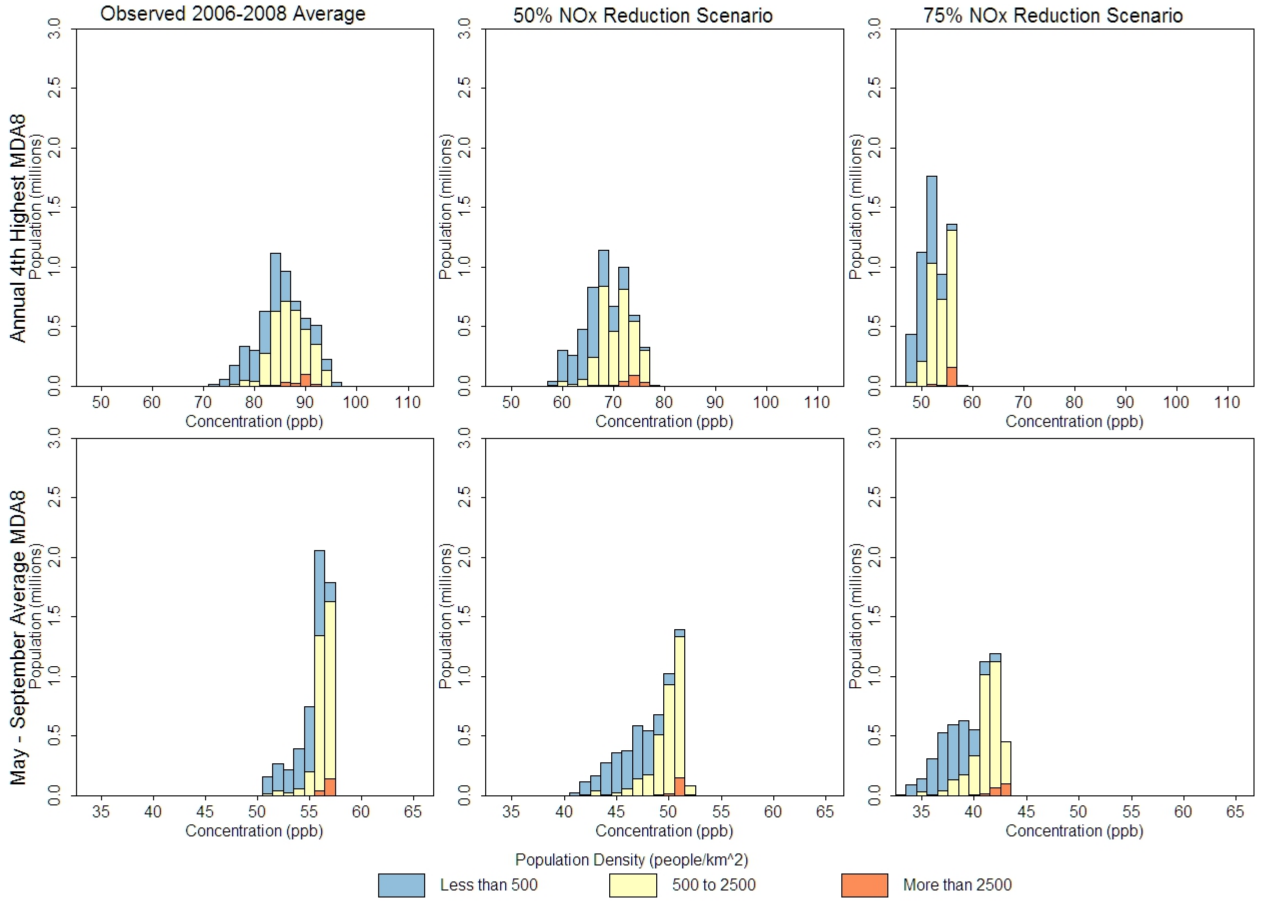

Figure S3. Histograms showing population living in Atlanta locations with various 2006-2008 average 4th highest MDA8 O3 (top panels, ppb) and May – September mean MDA8 O3 (bottom panels, ppb), for observed conditions (left panels), and predicted resulting conditions resulting from 50% U.S. NOx emissions reductions (center panels) and 75% U.S. NOx reductions (right panels). Colors show the breakdown of each histogram by population density.

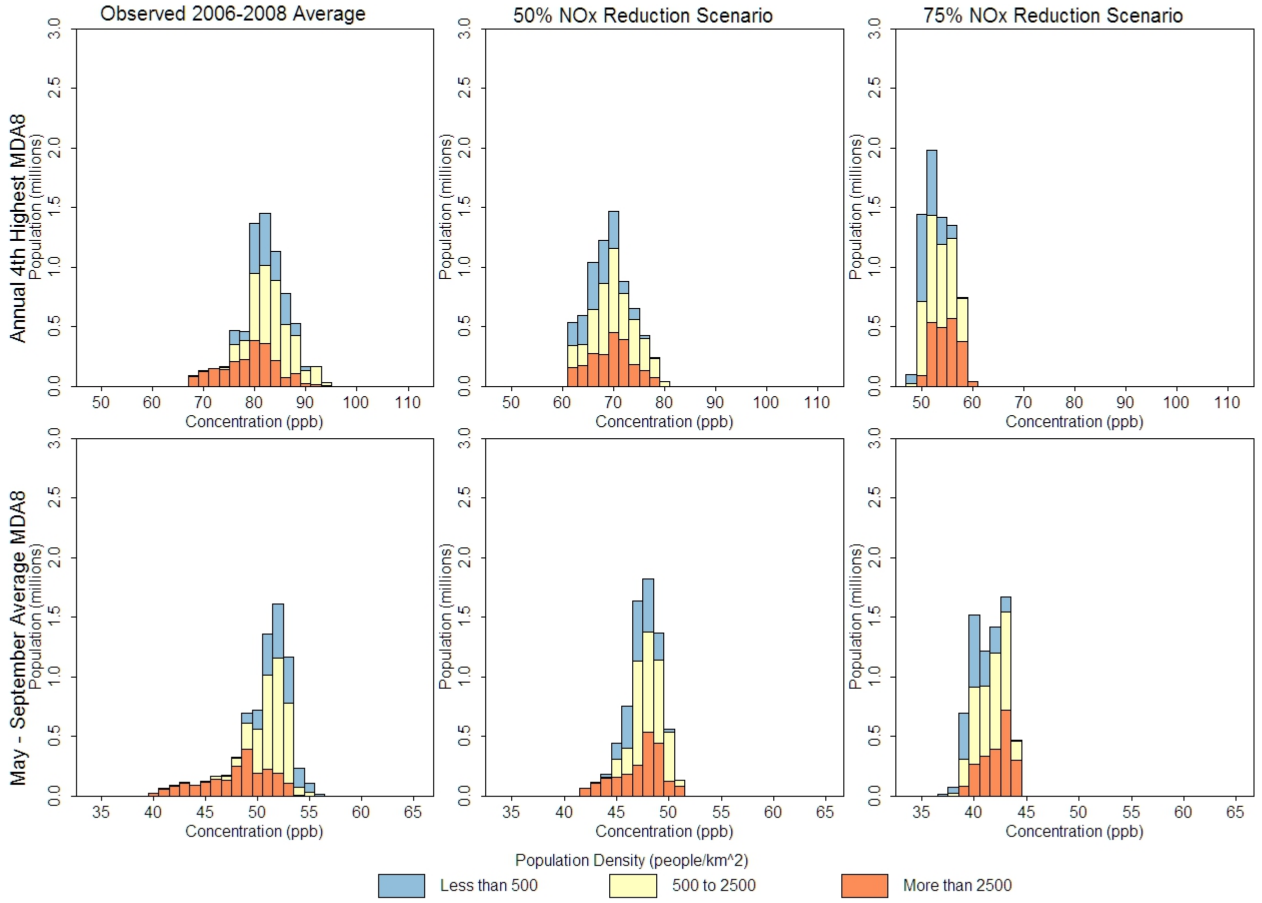

Figure S4. Histograms showing population living in Philadelphia locations with various 2006-2008 average 4th highest MDA8 O3 (top panels, ppb) and May – September mean MDA8 O3 (bottom panels, ppb), for observed conditions (left panels), and predicted resulting conditions resulting from 50% U.S. NOx emissions reductions (center panels) and 75% U.S. NOx reductions (right panels). Colors show the breakdown of each histogram by population density.

Environ Health Perspect
DOI: 10.1289/EHP190

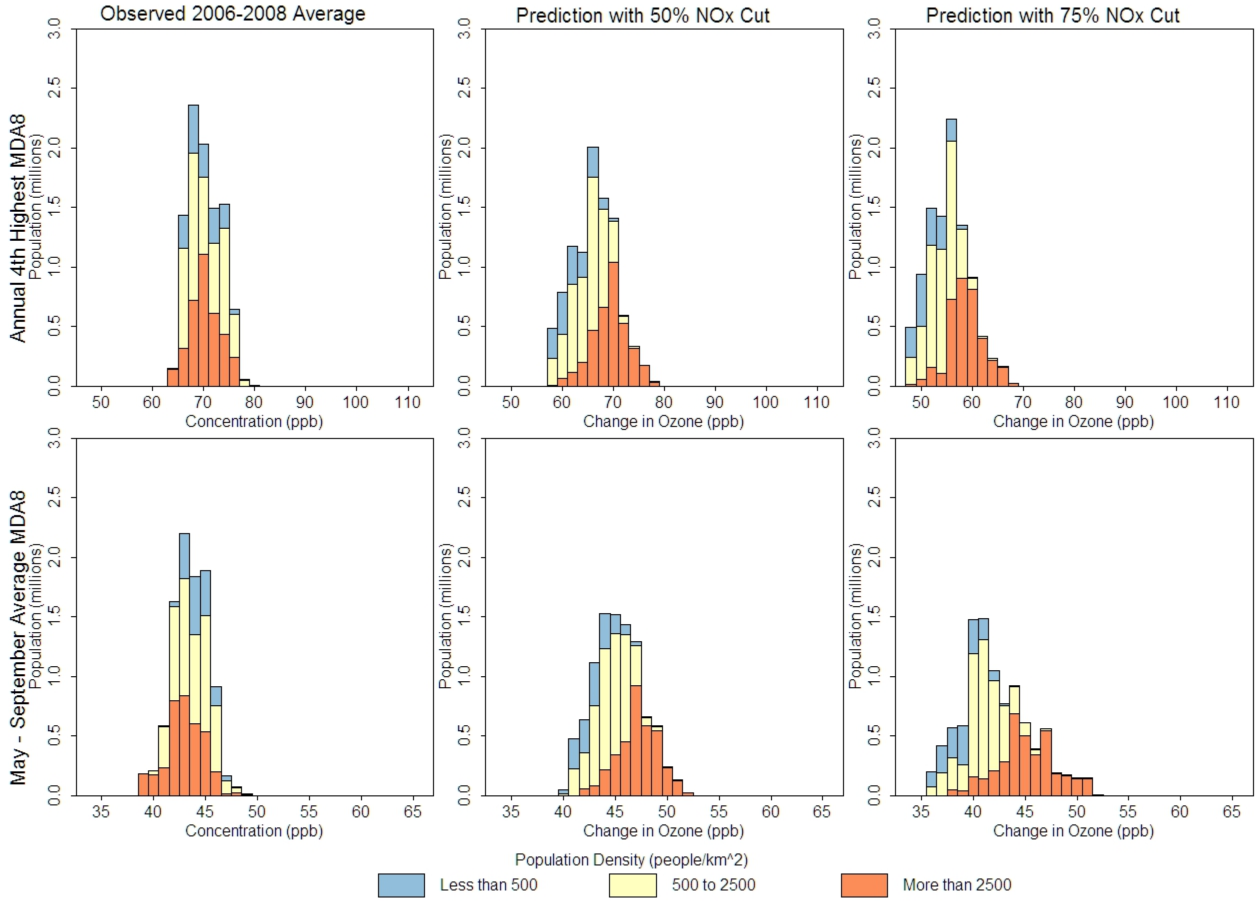

Figure S5. Histograms showing population living in Chicago locations with various 2006-2008 average 4th highest MDA8 O3 (top panels, ppb) and May – September mean MDA8 O3 (bottom panels, ppb), for observed conditions (left panels), and predicted resulting conditions resulting from 50% U.S. NOx emissions reductions (center panels) and 75% U.S. NOx reductions (right panels). Colors show the breakdown of each histogram by population density.

DOI: 10.1289/EHP190

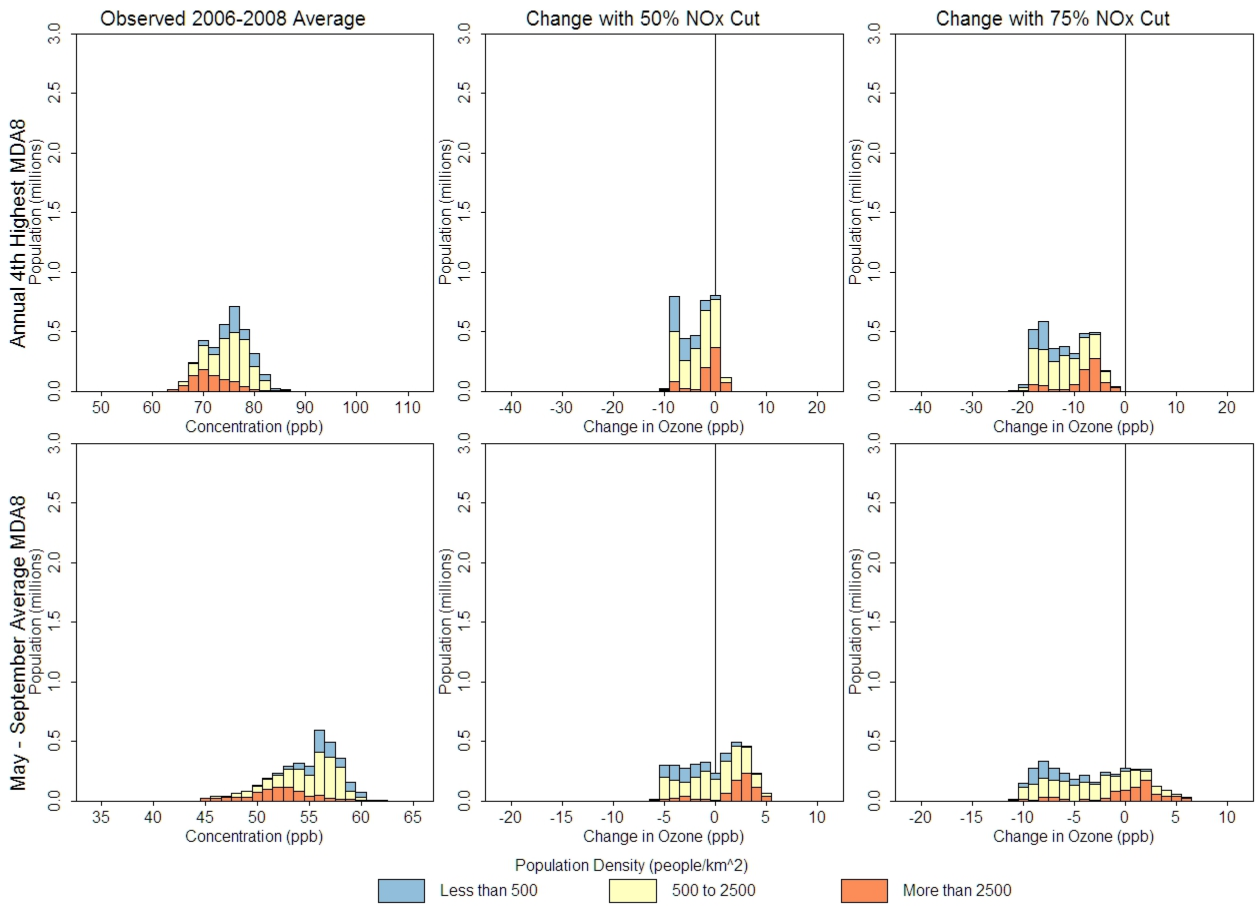

Figure S6. Histograms showing population living in Denver locations with various 2006-2008 average 4th highest MDA8 O3 (top panels, ppb) and May – September mean MDA8 O3 (bottom panels, ppb), for observed conditions (left panels), and predicted changes resulting from 50% U.S. NOx emissions reductions (center panels) and 75% U.S. NOx reductions (right panels). Colors show the breakdown of each histogram by population density.

DOI: 10.1289/EHP190

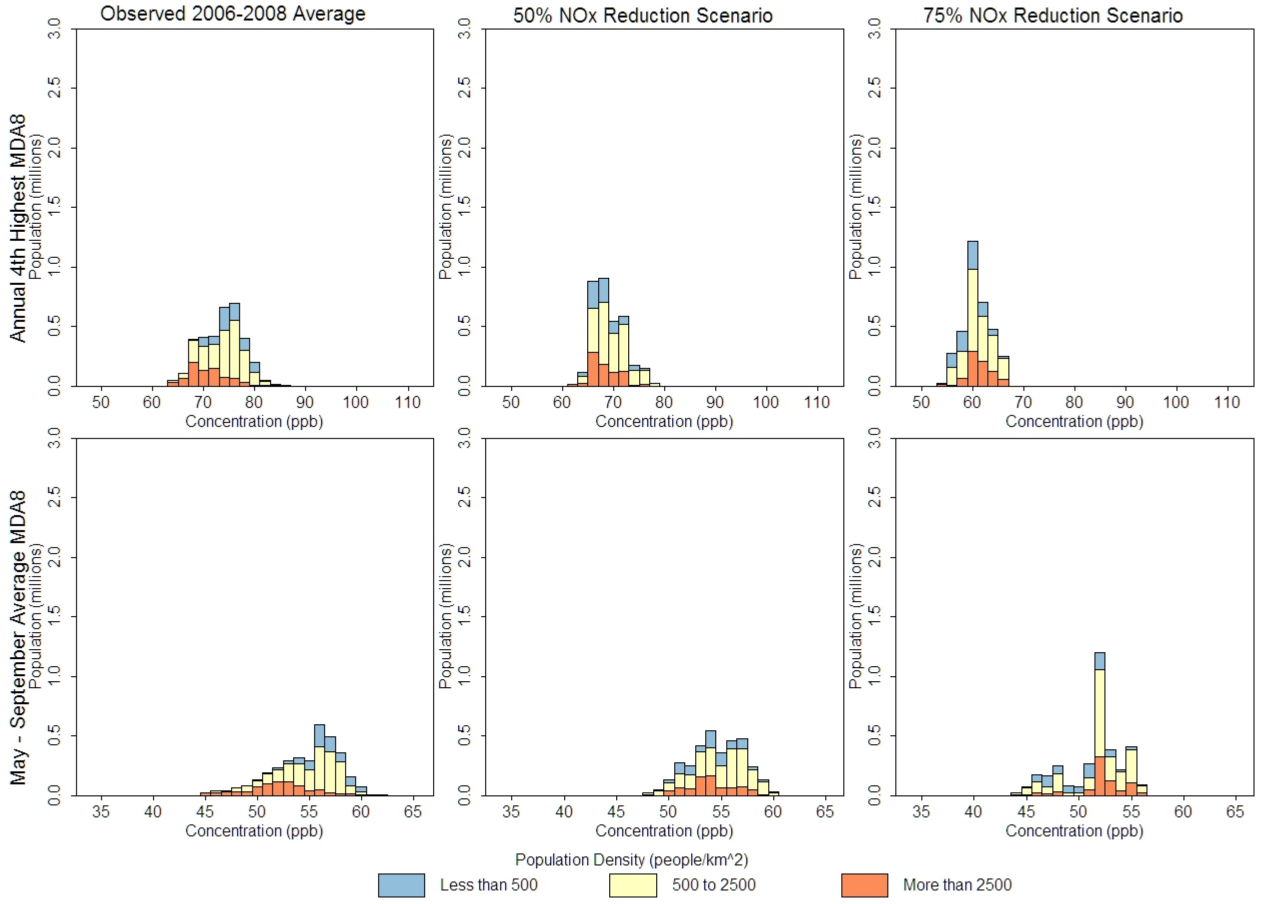

Figure S7. Histograms showing population living in Denver locations with various 2006-2008 average 4th highest MDA8 O3 (top panels, ppb) and May – September mean MDA8 O3 (bottom panels, ppb), for observed conditions (left panels), and predicted resulting conditions resulting from 50% U.S. NOx emissions reductions (center panels) and 75% U.S. NOx reductions (right panels). Colors show the breakdown of each histogram by population density.

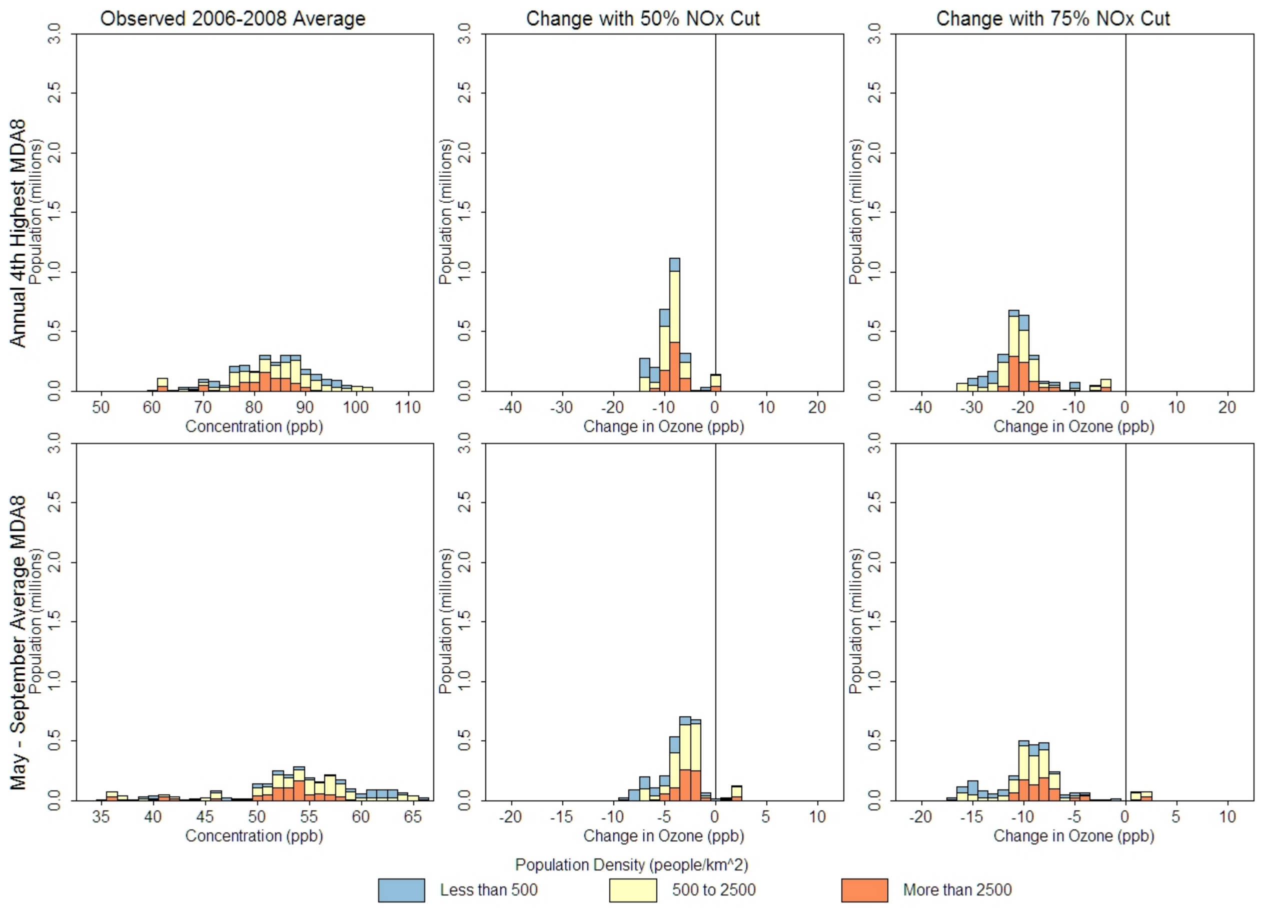

Figure S8. Histograms showing population living in Sacramento locations with various 2006-2008 average 4th highest MDA8 O3 (top panels, ppb) and May – September mean MDA8 O3 (bottom panels, ppb), for observed conditions (left panels), and predicted changes resulting from 50% U.S. NOx emissions reductions (center panels) and 75% U.S. NOx reductions (right panels). Colors show the breakdown of each histogram by population density.

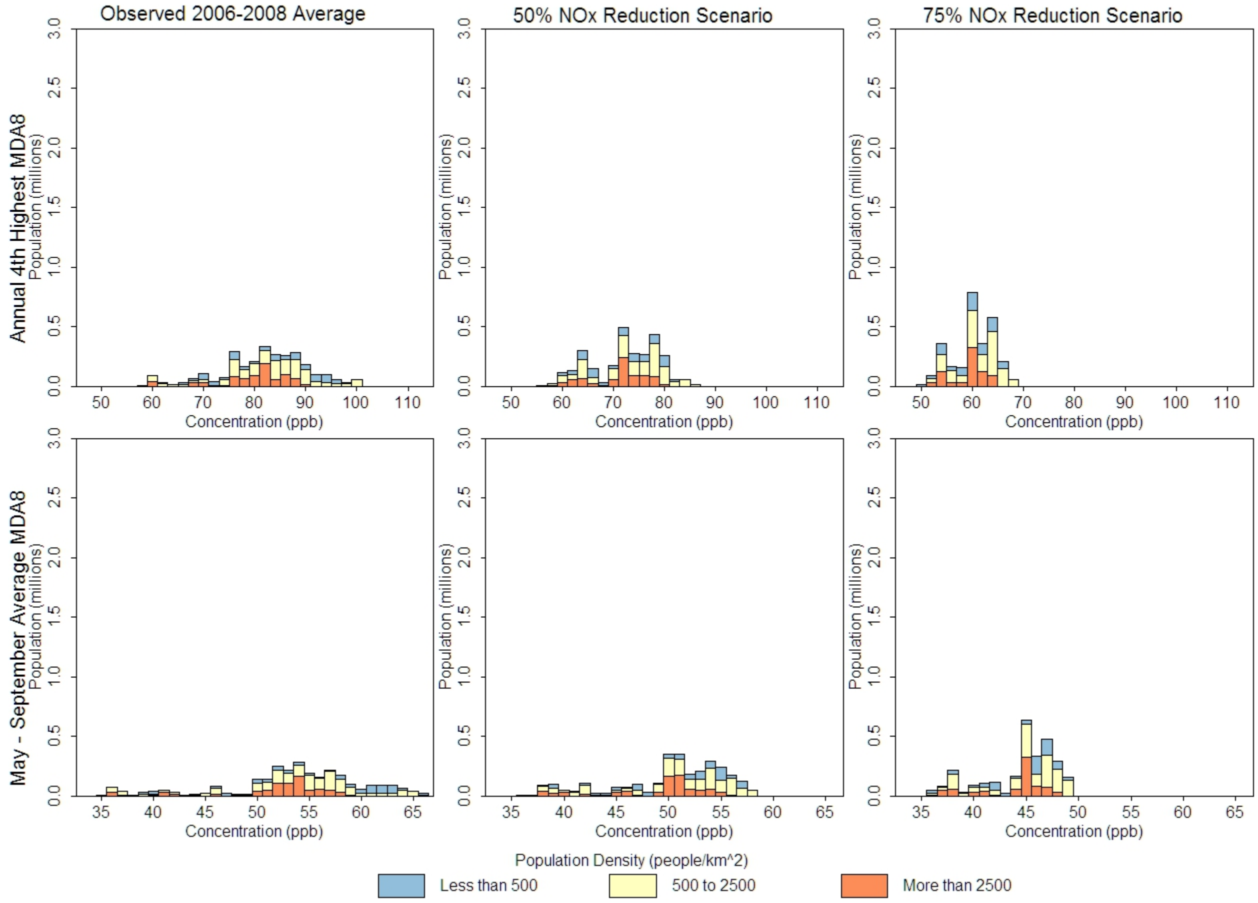

Figure S9. Histograms showing population living in Sacramento locations with various 2006-2008 average 4th highest MDA8 O3 (top panels, ppb) and May – September mean MDA8 O3 (bottom panels, ppb), for observed conditions (left panels), and predicted resulting conditions resulting from 50% U.S. NOx emissions reductions (center panels) and 75% U.S. NOx reductions (right panels). Colors show the breakdown of each histogram by population density.

Environ Health Perspect
DOI: 10.1289/EHP190

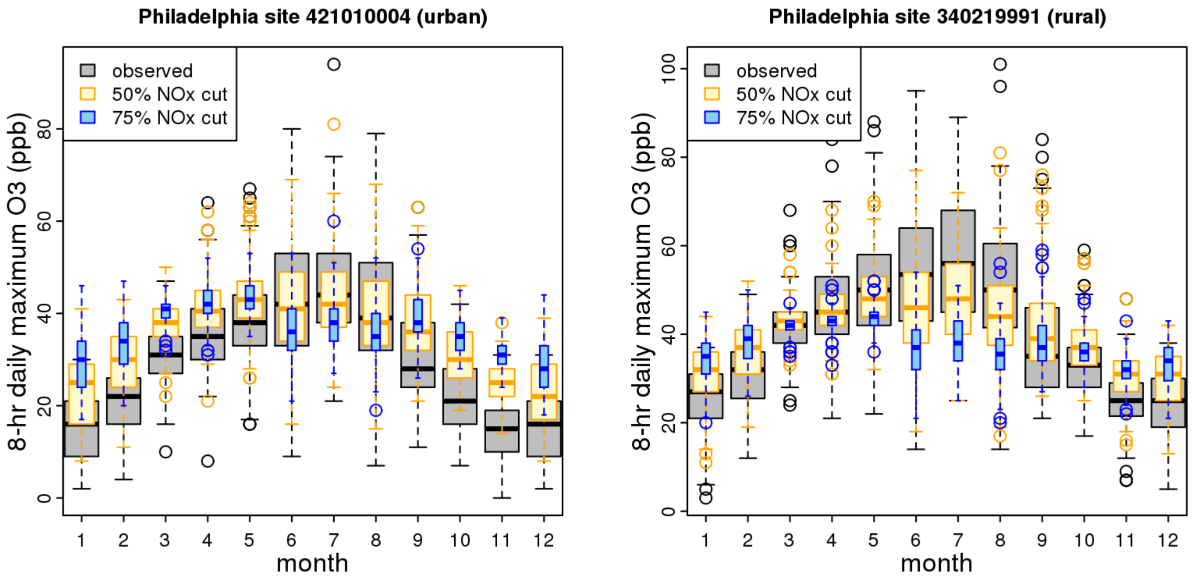

Figure S10. Distribution of 8-hr daily maximum O3 concentrations in Philadelphia by month at an urban and a rural monitoring site. Gray boxes show observed distribution. Yellow and blue boxes show predicted distribution after 50% and 75% reductions in US anthropogenic NOx emissions respectively. Horizontal bars show medial values, boxes outline the interquartile range, whiskers outline 1.5 times the interquartile range, and dots show outlier values.

DOI: 10.1289/EHP190

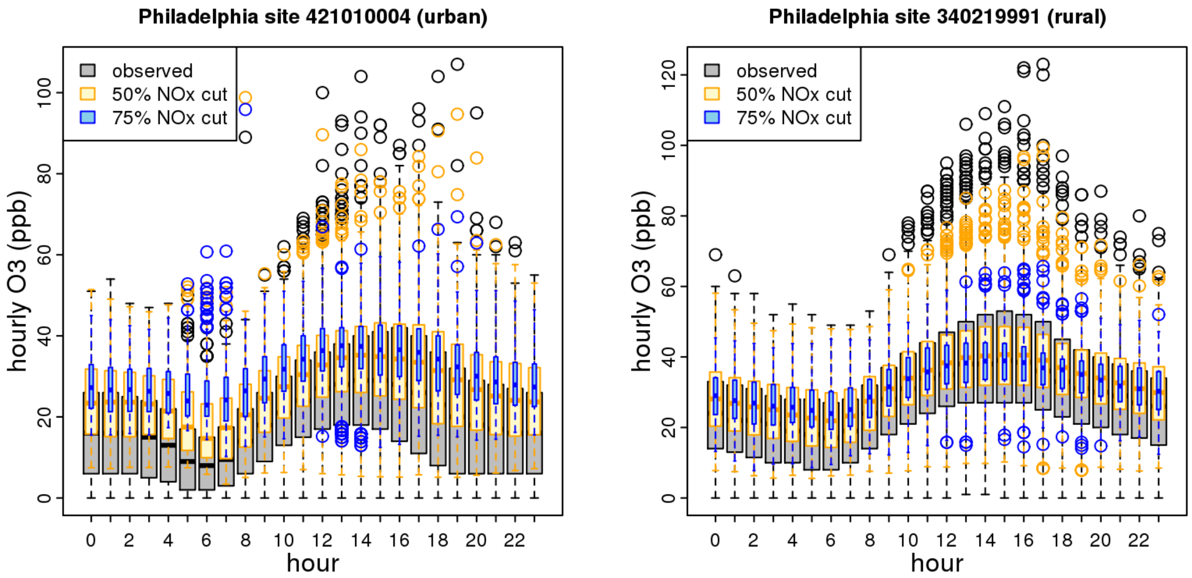

Figure S11. Distribution of hourly O3 concentrations in Philadelphia by hour of the day at an urban and a rural monitoring site. Gray boxes show observed distribution. Yellow and blue boxes show predicted distribution after 50% and 75% reductions in US anthropogenic NOx emissions respectively. Horizontal bars show medial values, boxes outline the interquartile range, whiskers outline 1.5 times the interquartile range, and dots show outlier values.

Environ Health Perspect
DOI: 10.1289/EHP190

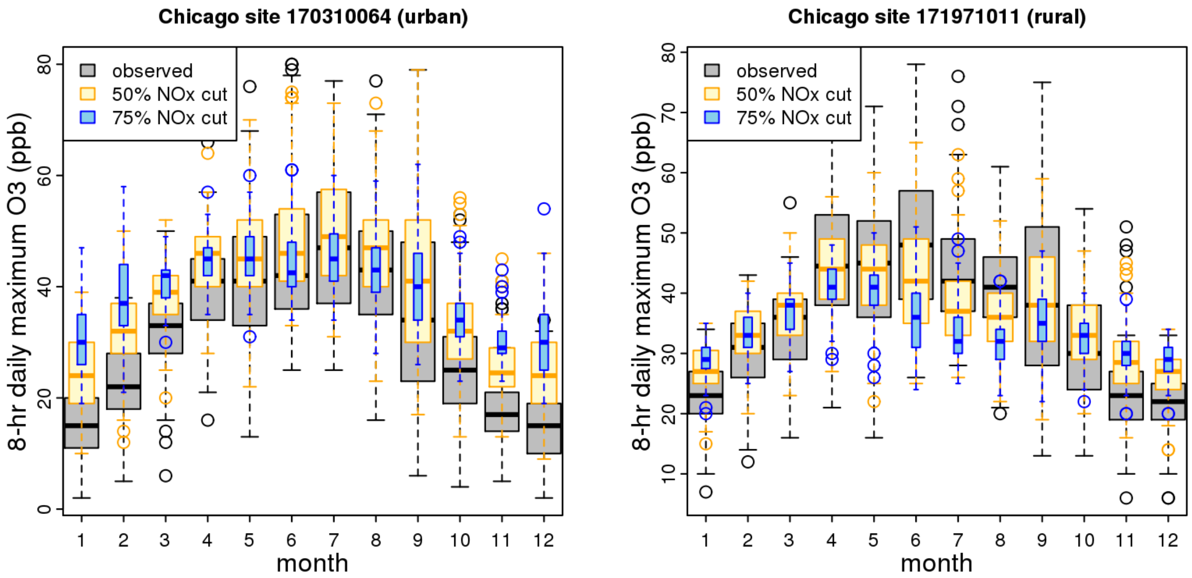

Figure S12. Distribution of 8-hr daily maximum O3 concentrations in Chicago by month at an urban and a rural monitoring site. Gray boxes show observed distribution. Yellow and blue boxes show predicted distribution after 50% and 75% reductions in US anthropogenic NOx emissions respectively. Horizontal bars show medial values, boxes outline the interquartile range, whiskers outline 1.5 times the interquartile range, and dots show outlier values.

Environ Health Perspect
DOI: 10.1289/EHP190

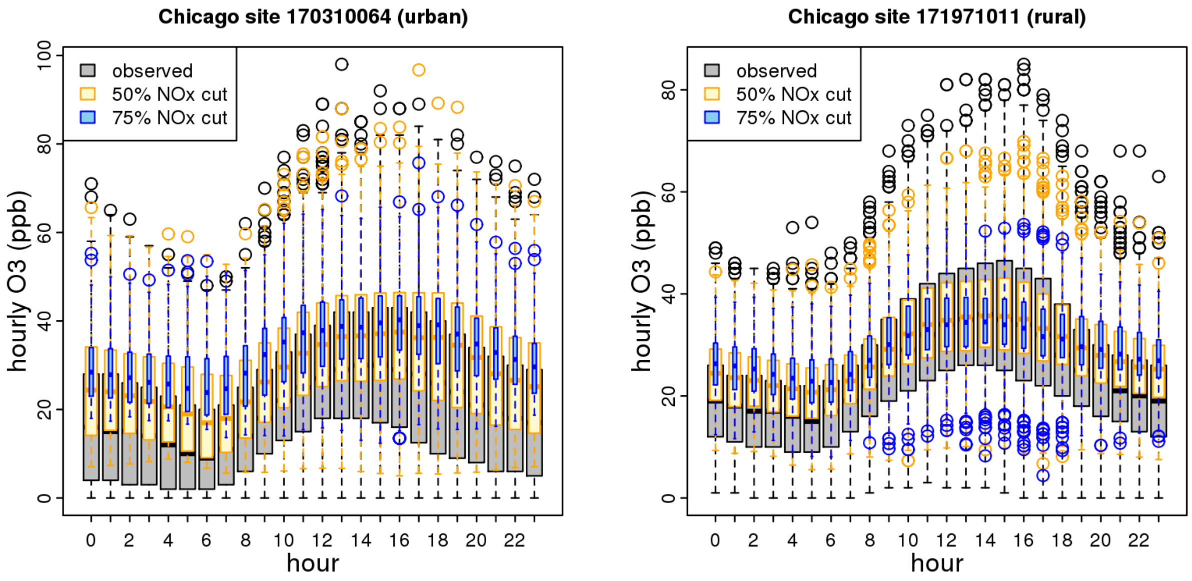

Figure S13. Distribution of hourly O3 concentrations in Chicago by hour of the day at an urban and a rural monitoring site. Gray boxes show observed distribution. Yellow and blue boxes show predicted distribution after 50% and 75% reductions in US anthropogenic NOx emissions respectively. Horizontal bars show medial values, boxes outline the interquartile range, whiskers outline 1.5 times the interquartile range, and dots show outlier values.

DOI: 10.1289/EHP190

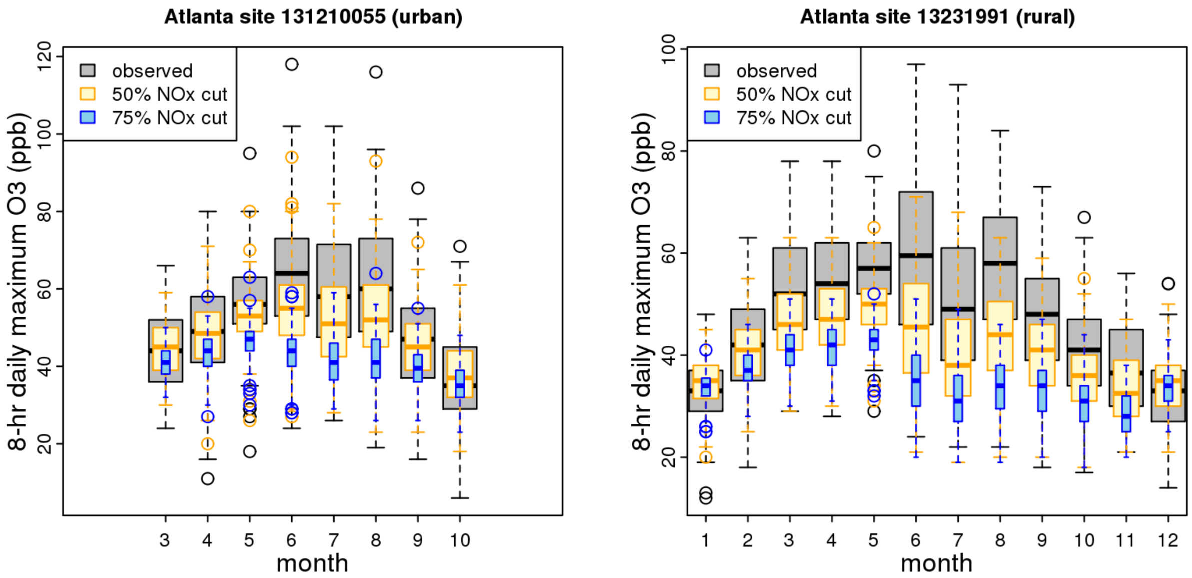

Figure S14. Distribution of 8-hr daily maximum O3 concentrations in Atlanta by month at an urban and a rural monitoring site. Gray boxes show observed distribution. Yellow and blue boxes show predicted distribution after 50% and 75% reductions in US anthropogenic NOx emissions respectively. Horizontal bars show medial values, boxes outline the interquartile range, whiskers outline 1.5 times the interquartile range, and dots show outlier values.

Environ Health Perspect
DOI: 10.1289/EHP190

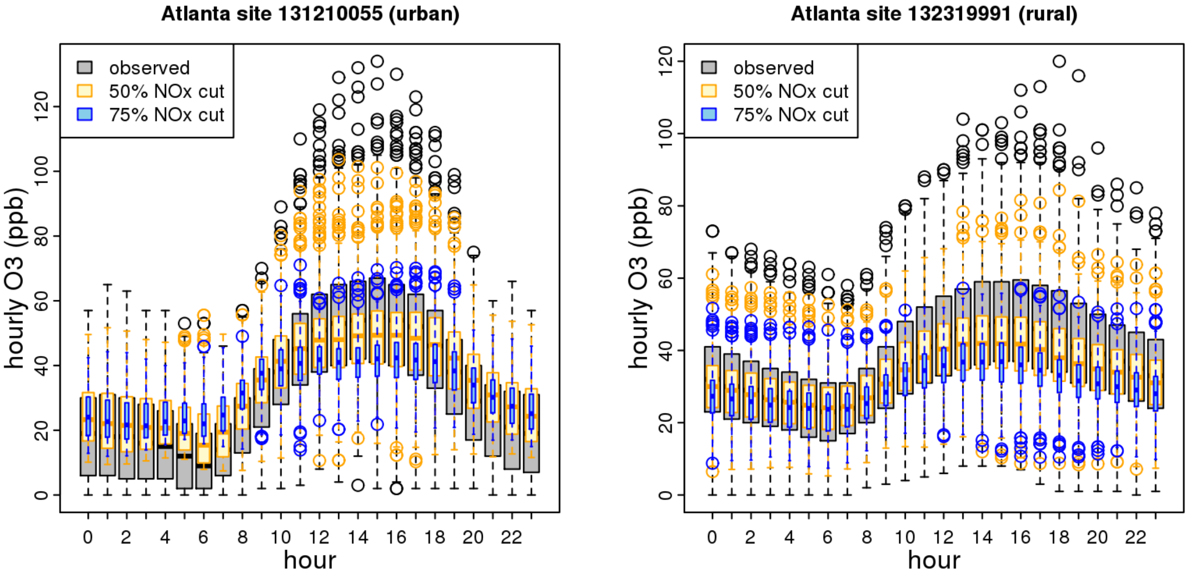

Figure S15. Distribution of hourly O3 concentrations in Atlanta by hour of the day at an urban and a rural monitoring site. Gray boxes show observed distribution. Yellow and blue boxes show predicted distribution after 50% and 75% reductions in US anthropogenic NOx emissions respectively. Horizontal bars show medial values, boxes outline the interquartile range, whiskers outline 1.5 times the interquartile range, and dots show outlier values.

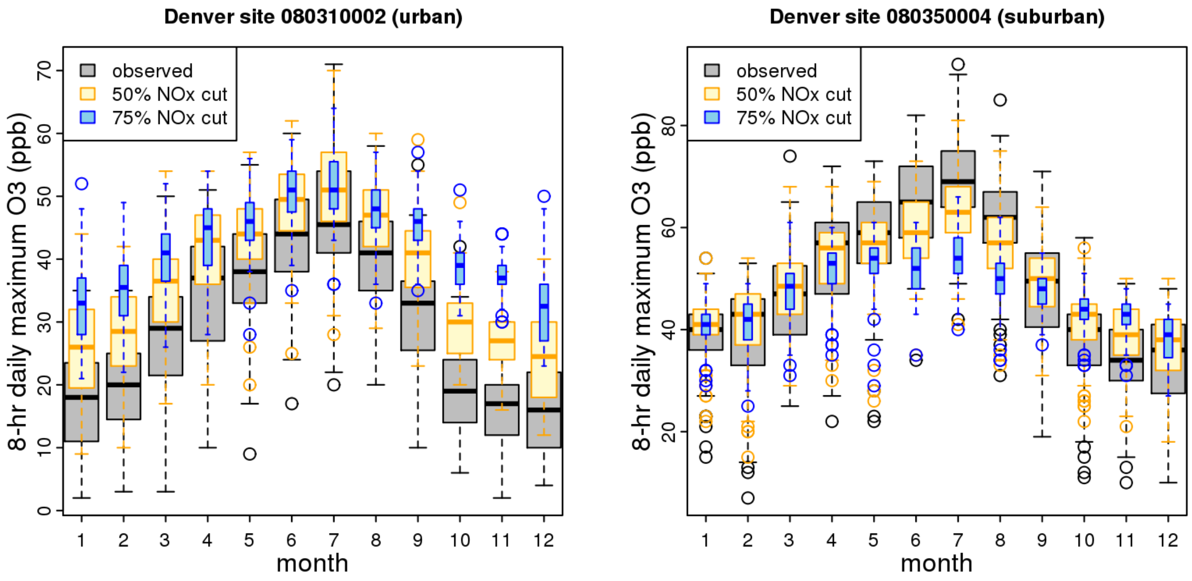

Figure S16. Distribution of 8-hr daily maximum O3 concentrations in Denver by month at an urban and a suburban monitoring site. Gray boxes show observed distribution. Yellow and blue boxes show predicted distribution after 50% and 75% reductions in US anthropogenic NOx emissions respectively. Horizontal bars show medial values, boxes outline the interquartile range, whiskers outline 1.5 times the interquartile range, and dots show outlier values.

DOI: 10.1289/EHP190

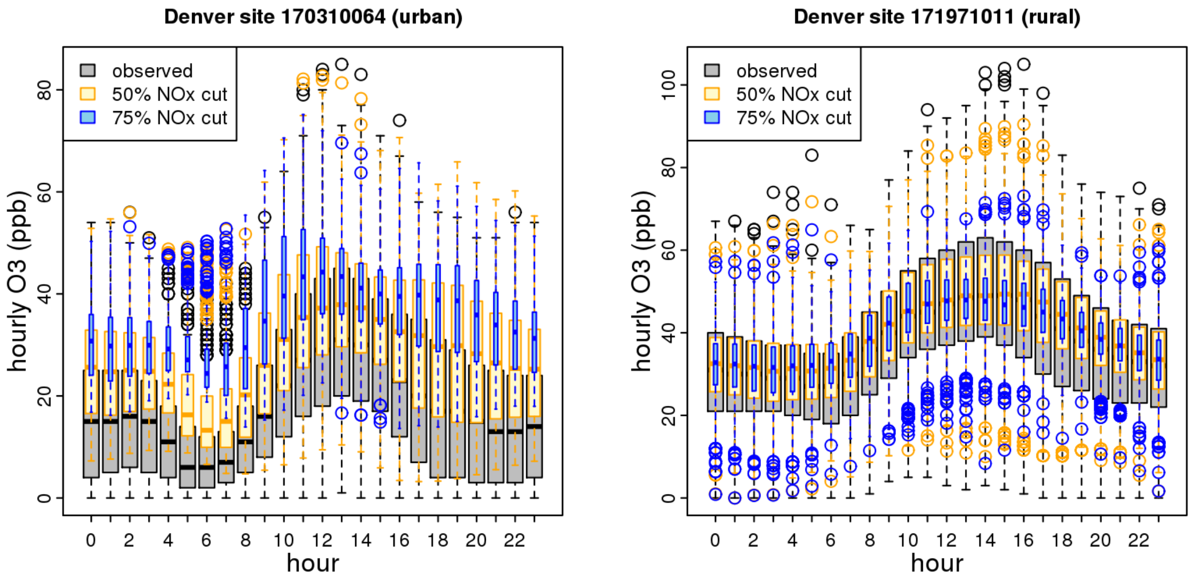

Figure S17. Distribution of hourly O3 concentrations in Denver by hour of the day at an urban and a suburban monitoring site. Gray boxes show observed distribution. Yellow and blue boxes show predicted distribution after 50% and 75% reductions in US anthropogenic NOx emissions respectively. Horizontal bars show medial values, boxes outline the interquartile range, whiskers outline 1.5 times the interquartile range, and dots show outlier values.

Environ Health Perspect
DOI: 10.1289/EHP190

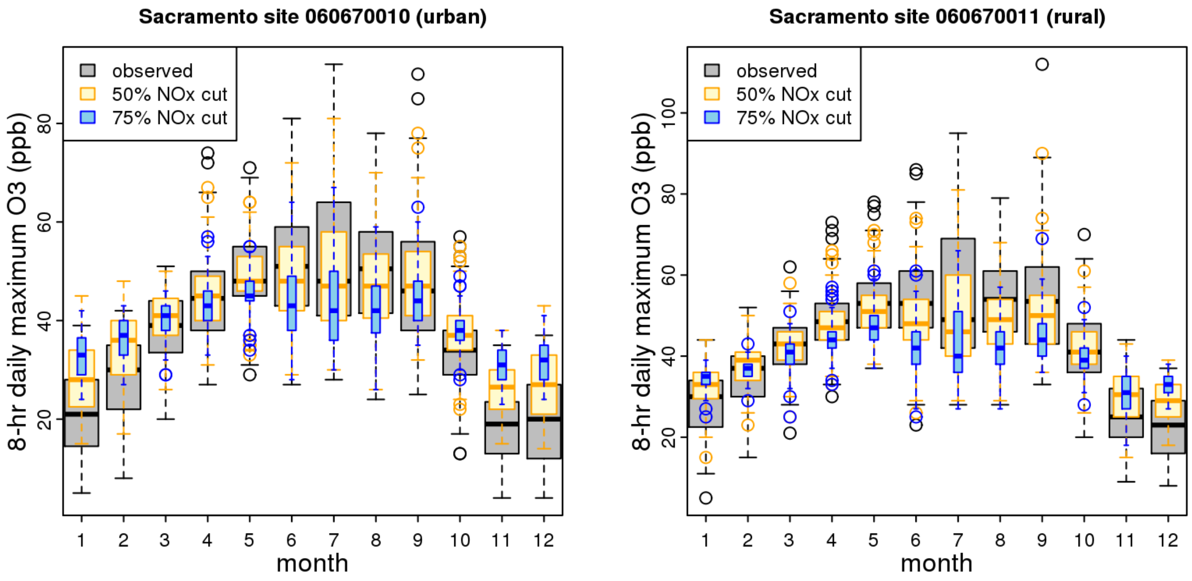

Figure S18. Distribution of 8-hr daily maximum O3 concentrations in Sacramento by month at an urban and a rural monitoring site. Gray boxes show observed distribution. Yellow and blue boxes show predicted distribution after 50% and 75% reductions in US anthropogenic NOx emissions respectively. Horizontal bars show medial values, boxes outline the interquartile range, whiskers outline 1.5 times the interquartile range, and dots show outlier values.

Environ Health Perspect
DOI: 10.1289/EHP190

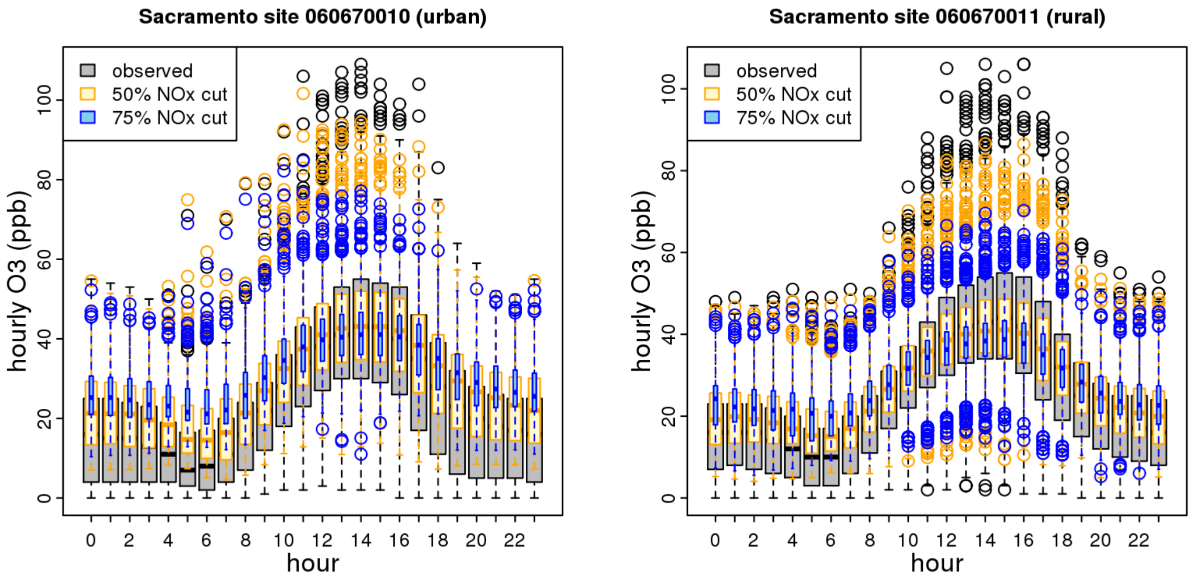

Figure S19. Distribution of hourly O3 concentrations in Sacramento by hour of the day at an urban and a rural monitoring site. Gray boxes show observed distribution. Yellow and blue boxes show predicted distribution after 50% and 75% reductions in US anthropogenic NOx emissions respectively. Horizontal bars show medial values, boxes outline the interquartile range, whiskers outline 1.5 times the interquartile range, and dots show outlier values.

DOI: 10.1289/EHP190

Supplemental References:

Akhtar F, Henderson B, Appel W, Napelenok S, Hutzell B, Pye H, et al. 2012. Multiyear Boundary Conditions for CMAQ 5.0 from GEOS-Chem with Secondary Organic Aerosol Extensions, 11th annual Community Modeling and Analysis System conference, Chapel Hill, NC, October 2012.

Foley KM, Roselle SJ, Appel KW, Bhave PV, Pleim JE, Otte TL, et al. 2010. Incremental testing of the Community Multiscale Air Quality (CMAQ) modeling system version 4.7. Geoscientific Model Development 3:205-226.

Gery MW, Whitten GZ, Killus JP, Dodge MC. 1989. A photochemical kinetics mechanism for urban and regional scale computer modeling. Journal of Geophysical Research-Atmospheres 94:12925-12956.

Henderson BH, Akhtar F, Pye HOT, Napelenok SL, Hutzell WT. 2013. A database and tool for boundary conditions for regional air quality modeling: description and evaluation. Geoscientific Model Development Discussions 6:4665-4704.

Skamarock WC, Klemp JB, Dudhia J, Gill DO, Barker DM, Dudia MG, et al. 2008. A Description of the Advanced Research WRF Version 3.

U.S. Environmental Protection Agency (EPA). 2012a. Air Quality Modeling Technical Support Document for the Regulatory Impact Analysis for the Revisions of the National Ambient Air Quality Standard for Particulate Matter, Research Triangle Park, NC. Available at: http://www.epa.gov/ttn/naaqs/standards/pm/data/201212aqm.pdf

DOI: 10.1289/EHP190

U.S. Environmental Protection Agency (EPA). 2012b. Technical Support Document: Preparation of Emissions Inventories of the Version 5, 2007-based Platform, Research Triangle Park, NC.

U.S. Environmental Protection Agency (EPA). 2014. Health Risk and Exposure Assessment for Ozone, Final Report, Research Triangle Park, NC, EPA-452/R-14-004a. http://www.epa.gov/ttn/naaqs/standards/ozone/s_o3_2008_rea.html

Yantosca B. 2004. GEOS-CHEMv7-01-02 User's Guide, Atmospheric Chemistry Modeling Group, Harvard University, Cambridge, MA.

Yarwood G, Rao S, Yocke M, Whitten GZ. 2005. Updates to the Carbon Bond chemical mechanism: CB05. Final Report to the US EPA, RT-0400675: http://www.camx.com/publ/pdfs/CB05_Final_Report_120805.pd
